# Iodine-mediated hydration of alkynes on keto-functionalized scaffolds: mechanistic insight and the regiospecific hydration of internal alkynes

**DOI:** 10.3762/bjoc.15.265

**Published:** 2019-11-14

**Authors:** Zachary Lee, Brandon R Jones, Nyochembeng Nkengbeza, Michael Phillips, Kayla Valentine, Alexis Stewart, Brandon Sellers, Nicholas Shuber, Karelle S Aiken

**Affiliations:** 1 Chemistry & Biochemistry, Georgia Southern University, PO Box 8064, Statesboro, GA 30460, USA

**Keywords:** α-iodo intermediate, internal alkyne, iodine-mediated hydration, neighboring group participation, regiospecific hydration

## Abstract

An iodine-mediated hydration reaction of alkynes serves as a green alternative to metal-catalyzed procedures. Previous work has shown that this method works well with terminal alkynes on keto-functionalized scaffolds, including 1,3-dicarbonyls and their heteroatom analogues. It was hypothesized that the reaction proceeds through a 5-*exo-dig* neighboring group participation (NGP) cyclization and an α-iodo intermediate. The work described herein probes the existence of the intermediate through NMR investigations and explores the scope of the hydration process with internal alkynes. The NMR experiments confirm the existence of the α-iodo intermediate, and methodology studies demonstrate that alkyl-capped, asymmetric, internal alkynes undergo a regiospecific hydration, also via the 5-*exo-dig* NGP pathway.

## Findings

The Kucherov reaction is one of the most well-known approaches for the hydration of alkynes. This reaction, originally published in 1881, requires the use of mercury(II) salts in catalytic quantities [1–2]. Countless studies have shown that mercury-based compounds are extremely harmful to environmental and biological systems [3]. In light of mercury’s toxicity, the synthetic community has given much attention to the use of less harmful reagents. Success has been obtained with many other transition metal salts, for example, palladium(II) [4–5], rhodium(III) [6], copper(II) [7], silver(I) [8], and gold(I)/(III) [9–12]. While less toxic than mercury, such catalysts are still environmental hazards and tend to be costlier [13–14]. The iodine-mediated hydration described herein is a viable solution to these issues [15–16]. This metal-free method produces innocuous, water-soluble byproducts, such as iodide and sulfate salts, and uses inexpensive molecular iodine to facilitate the hydration. Moreover, unlike other metal-free alkyne hydration procedures, heat and strong acids are not required [17–20]. The reaction proceeds under mild conditions at room temperature.

**Scheme 1:**
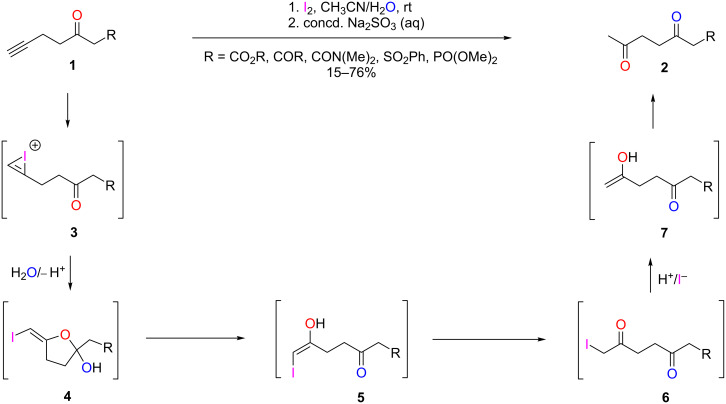
Proposed mechanism for the iodine-mediated hydration of terminal alkynes **1** [15].

Previous work by our group has shown that terminal alkynes on keto scaffolds undergo iodine-mediated hydration to form 1,4-diketo products **2** (Scheme 1) [15]. At that time, our studies revealed that a keto group must be present in the substrate, as the hydration requires neighboring group participation for 5-*exo-dig* cyclization from **3** to **4**. It was further hypothesized that the reaction continues through an α-iodo intermediate **6**, a tautomer of **5**, from which deiodination followed by tautomerization of **7** produces the final product **2**.

In the studies described herein, NMR experiments were used to probe the existence of an α-iodo intermediate **9**, and methodology work was used to investigate the outcome of the reaction using alkyl-capped, asymmetric, internal alkynes **11**.

The NMR investigations utilized different electrophilic iodine sources and the β-keto sulfone **8**, which allowed for detailed analysis of the reaction pathway. Compound **8**, having a relatively long reaction time of 6.5 h, was chosen for this study because its rate of reaction fitted the NMR timescale [15]. It should also be noted that the NMR experiments were performed in NMR tubes without stirring. As such, the reactions were much slower as compared to what has been reported for the larger-scale processes.

**Figure 1:**
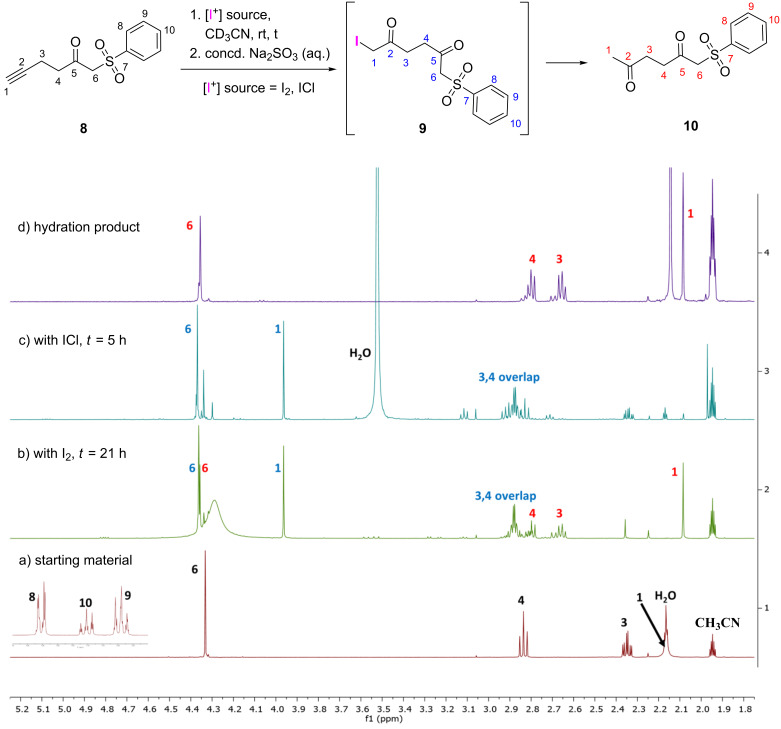
^1^H NMR investigations on the iodine-mediated hydration of **8** (the range of 1.75–5.25 ppm is displayed). a) Spectrum of starting material **8**. b) Spectrum of **8** upon treatment with two equivalents of I_2_. c) Spectrum of **8** upon treatment with two equivalents of ICl. d) Spectrum of the hydration product **10**. Figure 1a inset shows the phenyl region in the range of 7.55–8.00 ppm.

Initial attempts to observe the intermediate **9** involved the treatment of **8** with two equivalents of molecular iodine in deuterated acetonitrile (Figure 1a and Figure 1b). Significant changes in the ^1^H NMR spectrum of **8** were observed after 21 h, where new signals emerged and the substrate’s resonances disappeared. However, signals for **10** were also observed, suggesting that as soon as the intermediate **9** was formed, it was converted to the final hydration product **10**. Quenching of this reaction mixture cleanly resulted in the generation of **10** (Figure 1d). Changes in the resonances for the phenyl protons, H-8, H-9, and H-10, if any, were indiscernible.

**Figure 2:**
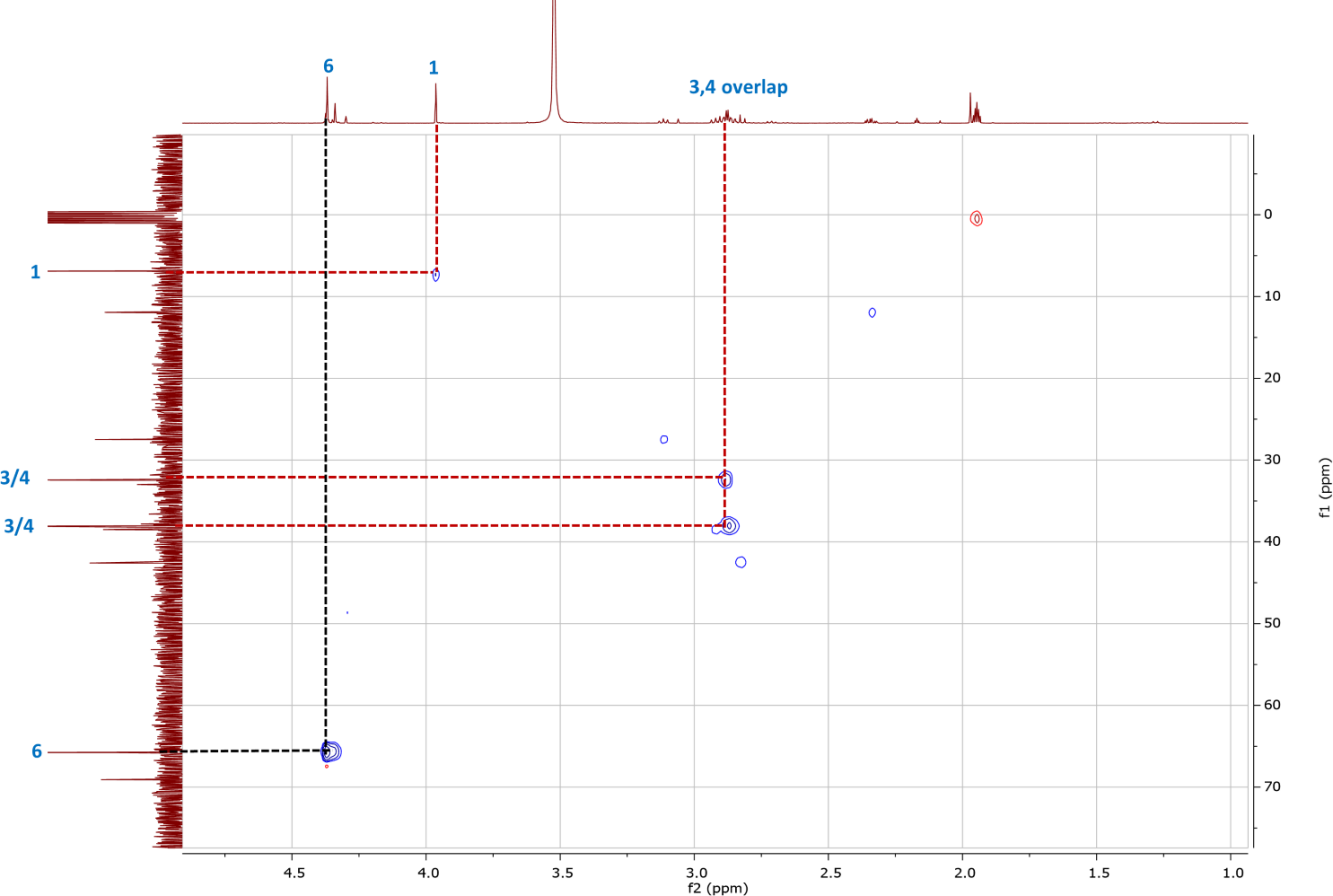
^1^H—^13^C HSQC spectrum for α-iodo intermediate **9** in CD_3_CN in the range of 0.90–5.00 ppm (for ^1^H NMR signals) and −1.0–77.0 ppm (for ^13^C NMR signals), respectively.

To increase the lifetime of the intermediate **9** so that more detailed analyses could be performed, the iodonium source was changed from I_2_ to ICl (Figure 1c). The chloride anion produced using ICl is less nucleophilic than iodide released from molecular iodine. As such, it was thought that the deiodination of intermediate **9** would proceed at a slower rate with Cl^−^ and hence, the intermediate would be longer lasting. This proved true: after five hours in the presence of two equivalents of ICl, signals at 2.85–2.93 ppm, 3.96 ppm, and 4.36 ppm, in addition to the phenyl protons, were observed, without any evidence of **10** being present. Based on literature reports for α-iodo ketones [21–22], it was clear that the signal at 3.96 ppm corresponded to H-1 in **9**. This was substantiated by a ^1^H,^13^C-HSQC experiment, which showed the signal corresponding to C-1 at 6.92 ppm in the ^13^C NMR spectrum, upfield from those for C-3 and C-4 (Figure 2). This relatively low ^13^C chemical shift was evidenced to be a result of the shielding effect of the iodo substituent on C-1. Similarities in the signals observed in Figure 1b and Figure 1c confirmed that the I_2_- and ICl-mediated hydrations occurred via the same intermediate. Furthermore, quenching of the ICl-facilitated reaction also resulted in the formation of compound **10**.

Importantly, the existence of the α-iodo intermediate **9** holds promise for further development of the reaction. The iodo group is an excellent leaving group and therefore, the generation of α-substituted hydration products through the addition of suitable nucleophiles to the reaction mixture is a strong possibility.

**Scheme 2:**
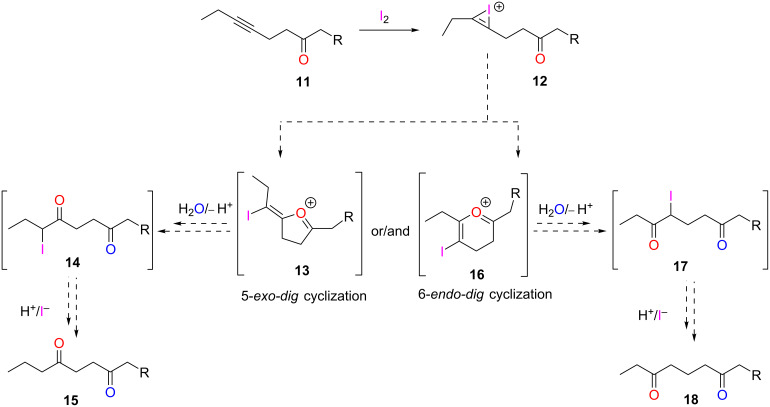
Possible outcomes of the iodine-mediated hydration of asymmetric, internal alkynes with neighboring group participation via the 5-*exo-dig* and 6-*endo-dig* pathway, respectively.

Having proven the existence of the α-iodo intermediate **9**, we turned our attention to investigating the reaction of internal alkynes **11** to examine the regioselectivity of the hydration process. Ester, sulfone, and phosphonate substrates were employed and, in all cases, the internal alkyne unit was capped by an ethyl group (Scheme 2). In general, asymmetric, internal alkynes capped by alkyl groups typically yield two ketone products, as the incorporation of the new oxo group can occur at either carbon atom of the triple bond. In fact, unless the substrate contains a carbocation-stabilizing group, such as a phenyl unit, hydration of asymmetric, internal alkynes results in a mixture of regioisomers [20,23–24]. Moreover, per Baldwin’s rules, with neighboring group participation, a 5-*exo-dig* (via **13** and **14**) or 6-*endo-dig* pathway (via **16** and **17**), or both, are possible outcomes for such alkynes [25]. The 5-*exo-dig* route would provide **15**, while the 6-*endo-dig* cyclization would result in diketone **18**.

**Scheme 3:**
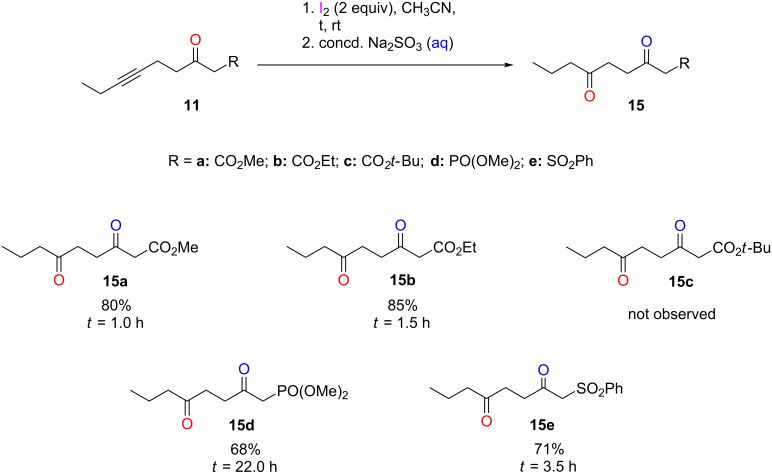
Iodine-mediated hydration of asymmetric, internal alkynes **11a**–**e**.

Significantly, the iodine-mediated hydration of all substrates **11** that were studied gave rise to one type of product, **15** (Scheme 3). This indicated that the reaction with alkyl-capped, internal alkynes occurred exclusively through the 5-*exo-dig* route, a regiospecific outcome. Accordingly, derivatives of **18** were never observed.

The methyl and ethyl esters **11a** and **11b** were subjected to the standard conditions, two equivalents of iodine in acetonitrile at room temperature [15]. Substrates **11a** and **11b** provided the hydration products in good yield, 80% for **15a** and 85% for **15b**. For the *tert*-butyl ester **11c**, multiple attempts were made with different variations, including adjustments in the reaction time, variations in the equivalents of iodine used, and the addition of water to facilitate the process. However, neither the starting material **11c** nor **15c** were ever recovered. In contrast, the phosphonate **11d** and the sulfone **11e** smoothly converted to **15d** and **15e** in yields of 68% and 71%, respectively. The hydration of the phosphonate required 22 h, while the reaction of the sulfone was complete in 3.5 h. Overall, successful outcomes were obtained for the phosphonate, sulfone, and methyl/ethyl ester substrates at room temperature under the mild reaction conditions employed.

## Conclusion

The studies described herein confirm the existence of an α-iodo intermediate in the iodine-mediated hydration of alkynes on a keto scaffold and also demonstrate that the reaction is regiospecific for alkyl-capped, asymmetric, internal alkynes **11**. The α-iodo intermediate **9** was observed using NMR spectroscopic investigations to monitor the conversion from alkyne to ketone in situ. Moreover, the use of ICl in the NMR studies, as opposed to I_2_, allowed for clear and unambiguous characterization of the intermediate **9**. With respect to the internal alkynes **11**, the reaction was regiospecific, occurring exclusively via a 5-*exo-dig* NGP pathway. Yields between 68% and 85% were obtained for **15** using the representative substrates **11**. This hydration process is amenable to methyl and ethyl esters, as well as phosphonate and sulfone analogues of the keto scaffold. Notably, the existence of the iodo intermediate points at the potential to synthesize α-substituted targets upon further development of the method.

## Supporting Information

File 1 Synthetic and analytical details for all compounds.
