# Supplementary material for: Iodine-mediated hydration of alkynes on keto-functionalized scaffolds: mechanistic insight and the regiospecific hydration of internal alkynes
Source: Beilstein J Org Chem. 2019 Nov 14;15:2747–52. doi: 10.3762/bjoc.15.265 (PMC6880791; doi:10.3762/bjoc.15.265)
Supplement: File 1 — Synthetic and analytical details for all compounds. [file Beilstein_J_Org_Chem-15-2747-s001.pdf]

BEILSTEIN JOURNAL OF ORGANIC CHEMISTRY

Supporting Information

for

Iodine-mediated hydration of alkynes on keto-functionalized scaffolds: mechanistic insight and the regiospecific hydration of internal alkynes

Zachary Lee, Brandon R. Jones, Nyochembeng Nkengbeza, Michael Phillips, Kayla Valentine, Alexis Stewart, Brandon Sellers, Nicholas Shuber and Karelle S. Aiken

Beilstein J. Org. Chem. **2019,** *15,* 2747–2752. doi:10.3762/bjoc.15.265

Synthetic and analytical details for all compounds

License and Terms: This is a supporting information file under the terms of the Creative Commons Attribution License (http://creativecommons.org/licenses/by/4.0). Please note that the reuse, redistribution and reproduction in particular requires that the authors and source are credited. The license is subject to the *Beilstein Journal of Organic Chemistry* terms and conditions: (https://www.beilstein-journals.org/bjoc)

Table of Contents

General experimental S2

Procedure for the iodine-mediated hydration S2

Synthesis of the keto alkyne scaffolds S5

C/H Correlatiom spectrum for **9** S8

^{1}H and ^{13}C NMR spectra for substrates and hydration products S11

High resolution mass spectra (negative ion mode) S29

References S31

I. General experimental:

Solvents used in reactions requiring air-free/dry conditions were obtained from a MBraun Manual Solvent Purification System and stored over 4 Å molecular sieves under nitrogen prior to use. Otherwise, unless noted, solvents for procedures performed under ambient conditions were obtained and used as is from commercial sources. Chromatography was performed with Selecto Scientific Si gel (particle size 100–200 µm). NMR spectra were recorded on a Agilent MR400DD2 spectrometer, with a multinuclear probe with two RF channels and variable temperature capability (^{1}H NMR: 400 MHz, ^{13}C NMR: 101 MHz). For NMR, the solvent was $CDCl_3$ and signals are reported in parts per million (ppm) relative to residual $CHCl_3$. Signals are described with multiplicity, singlet (s), doublet (d), triplet (t), triplet of doublet (td), quartet (q), sextet (sx) and multiplet (m); coupling constants (*J*; Hz) and integration. The accurate mass was acquired at Georgia State University Mass Spectrometry Facility using Waters QTof micro mass spectrometer with an ESI source in a negative mode. The accurate mass was measured in the presence of an internal standard.

II. Iodine-mediated hydration:

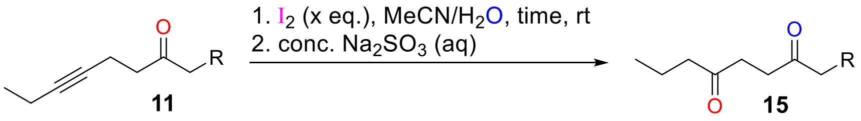

SUBSTRATE	R
**a**	CO_2Me
**b**	CO_2Et
**c**	CO_2*t*Bu
**d**	$PO(OMe)_2$
**e**	SO_2Ph

Scheme S1: Iodine-mediated hydration of the keto alkyne scaffolds

a. Representative procedure

Methyl 3,6-dioxononanoate (**15a**)

A vial equipped with a stirring bar was charged with methyl 3-oxonon-6-ynoate (**11a**, 91.1 mg, 0.5 mmol) dissolved in acetonitrile (10 mL) followed by the addition of iodine (253.8 mg,

1.0 mmol). The reaction mixture was stirred under ambient atmosphere at room temperature and monitored by TLC. Upon completion, after 1.0 hours, the reaction was quenched with concentrated aqueous sodium sulfite (5 mL) with vigorous stirring for thirty minutes. The resulting yellow suspension was extracted three times with ethyl acetate (10 mL). The combined ethyl acetate extract was washed with DI water (10 mL) followed by brine (10 mL) and, dried with sodium sulfate. The dried organic extract was concentrated under vacuum and the crude product was purified by flash column chromatography (10% ethyl acetate in hexanes followed by 15% ethyl acetate in hexanes) to provide **15a** as pale yellow oil (80.1 mg, 80%).^{1}**H NMR** (400 MHz, $CDCl_3$) δ 3.72 (s, 3H), 3.50 (s ,2H), 2.79 (t, *J* = 6.0 Hz, 2H), 2.69 (t, *J* = 6.0 Hz, 2H), 2.41 (t, *J* = 7.3 Hz, 2H), 1.59 (sx, *J* = 7.4 Hz, 2H)), 0.89 (t, *J* = 7.4 Hz, 3H); ^{13}**C NMR** (101 MHz, $CDCl_3$) δ 209.0, 201.5, 167.5, 52.3, 49.1, 44.6, 36.3, 36.1, 17.3, 13.7; **HRMS** (ESI, negative ion mode) *m/z*: $[M - H]^-$ calculated for $C_{10}H_{15}O_4$: 199.0970; Found: 199.0960.

Ethyl 3,6-dioxononanoate (**15b**). Synthesized from ethyl 3-oxonon-6-ynoate **11b** according to the procedure for **15a**, reaction time: 1.5 h; purification with 10% ethyl acetate in hexanes followed by 15% ethyl acetate in hexanes, orange oil, (90.5 mg, 85%). ^{1}**H NMR** (400 MHz, $CDCl_3$) δ 4.15 (q, *J* = 7.4 Hz, 2H), 3.47 (s, 2H), 2.78 (t, *J* = 6.8 Hz, 2H), 2.68 (t, *J* = 6.8 Hz, 2H), 2.40 (t, *J* = 7.3 Hz, 2H), 1.58 (sx, *J* = 7.4 Hz, 2H), 1.24 (t, *J* = 7.1 Hz, 3H), 0.88 (t, *J* = 7.4 Hz, 3H); ^{13}**C NMR** (101 MHz, $CDCl_3$) δ 208.9, 201.6, 167.1, 61.3, 49.3, 44.6, 36.3, 36.0, 17.2, 14.0, 13.6 **HRMS** (ESI, negative ion mode) *m/z*: $[M - H]^-$ calculated for $C_{11}H_{17}O_4$: 213.1127; Found: 213.1119.

1-(Dimethylphosphoryl)octane-2,5-dione (**15d**) Synthesized from dimethyl (2-oxooct-5-yn-1-yl)phosphonate **11d** according to the procedure for **15a**, reaction time: 22 h; purification with 100% ethyl acetate, orange oil, (85.6 mg, 68%). **^{1}H NMR** (400 MHz, $CDCl_3$) δ 3.75 (d, $^{3}J_{H\text{-}P}$ = 11.3 Hz, 6H), 3.12 (d, $^{2}J_{H\text{-}P}$ = 22.7 Hz, 2H), 2.84 (t, *J* = 6.1 Hz, 2H), 2.65 (t, *J* = 6.1 Hz, 2H), 2.39 (t, *J* = 74. Hz, 2H), 1.56 (sx, *J* = 7.4 Hz, 2H), 0.86 (t, *J* = 7.4 Hz, 3H); **^{13}C NMR** (101 MHz, $CDCl_3$) δ 209.1, 200.6 (d, $J_{C\text{-}P}$ = 6.2 Hz), 53.0 (d, $J_{C\text{-}P}$ = 6.5 Hz), 44.5, 41.2 (d, $J_{C\text{-}P}$ = 128.9 Hz), 37.5 (d, $J_{C\text{-}P}$ = 1.8 Hz), 36.0, 17.2, 13.6; **HRMS** (ESI, negative ion mode) *m/z*: $[M - H]^-$ calculated for $C_{10}H_{18}O_5P$: 249.0892; Found: 249.0883.

1-(Phenylsulfonyl)octane-2,5-dione (**15e**). Synthesized from 1-(phenylsulfonyl)oct-5-yn-2-one **11e** according to the procedure for **15a**, reaction time: 3.5 h; purification with 20% ethyl acetate in hexanes, orange oil, (100.3 mg, 71%). **^{1}H NMR** (400 MHz, $CDCl_3$) δ 7.88 (d, *J* = 7.9 Hz, 2H), 7.65 (tt, *J* = 7.4, 1.5 Hz, 1H), 7.55 (tt, *J* = 7.9, 1.5 Hz, 2H), 4.21 (s, 2H), 2.92 (t, *J* = 5.8 Hz, 2H), 2.69(t, *J* = 5.8 Hz, 2H), 2.37 (t, *J* = 7.3 Hz, 2H), 1.55 (sx, *J* = 7.4 Hz, 2H), 0.87 (t, *J* = 7.4 Hz, 3H); **^{13}C NMR** (101 MHz, $CDCl_3$) δ 208.6, 197.1, 138.8, 134.2, 129.3, 128.2, 67.2, 44.4, 37.7, 36.3, 17.2, 13.6; **HRMS** (ESI, negative ion mode) *m/z*: $[M - H]^-$ calculated for $C_{14}H_{17}O_4S$: 281.0848; Found: 281.0846.

III. Synthesis of the keto alkyne scaffolds:

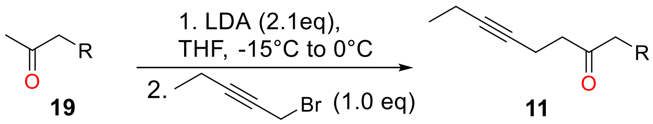

SUBSTRATE	R
**a**	CO_2Me
**b**	CO_2Et
**c**	CO_2tBu
**d**	$PO(OMe)_2$
**e**	SO_2Ph

Scheme S1: Synthesis of the keto alkyne scaffolds

a. Representative procedure

Methyl 3-oxonon-6-ynoate (**11a**)

An oven-dried round bottomed flask equipped with a stirring bar and nitrogen-inlet was charged with freshly distilled diisopropylamine (2.54 mL, 17.4 mmol) in dry THF (25.5 mL). The solution was cooled with an ice-water bath (0 °C) and treated with a drop-wise addition of *n*-butyllithium (2.5 M hexanes solution, 6.36 mL, 17.4 mmol). After 30 minutes, the resulting transparent, yellow solution was cooled with an ice–salt water bath (−15 °C) and methyl 3-oxobutanoate (0.82 mL, 7.57 mmol) was added drop-wise. The resulting solution had an intense orange-brown color. After 45 minutes, the reaction mixture was treated with drop-wise addition of 1-bromopent-2-yne (0.72 mL, 7.64 mmol). The reaction mixture was stirred overnight and allowed to warm to room temperature. The reaction was quenched with an aqueous solution of saturated ammonium chloride (10 mL) and extracted twice with ethyl acetate (50 mL). The combined organic extract was washed once with deionized (DI) water (10 mL) followed by brine (10 mL), and dried with sodium sulfate. The dried organic extract was concentrated under vacuum and the crude orange product was purified by flash column chromatography (20% ethyl acetate in hexanes) to provide **11a**, yellow oil, (745.0 mg, 54%). Compound **11a** is known [1]. **^{1}H NMR** (400

MHz, $CDCl_3$) δ 3.72 (s, 3H), 3.47 (s, 2H), 2.73 (t, *J* = 7.3 Hz, 2H), 2.43 (tt, *J* = 7.3, 2.4 Hz, 2H), 2.11 (qt, *J* = 7.5, 2.4 Hz, 2H), 1.07 (t, *J* = 7.5 Hz 3H); **^{13}C NMR** (101 MHz, $CDCl_3$) δ 201.0, 167.4, 89.4, 82.6, 52.4, 48.9, 42.3, 14.1, 13.3, 12.3.

Ethyl 3-oxonon-6-ynoate (**11b**). Synthesized from ethyl acetoacetate according to the procedure for **11a**; purification with 10% ethyl acetate in hexanes, yellow oil, (1.02 g, 52% based on 10 mmol ethyl acetoacetate). **^{1}H NMR** (400 MHz, $CDCl_3$) δ 4.19 (q, *J* = 7.1 Hz, 3H), 3.45 (s, 2H), 2.73 (t, *J* = 7.3 Hz, 2H), 2.42 (t, *J* = 7.3, 2.4Hz, 2H), 2.12 (qt, *J* = 7.5, 2.4 Hz, 2H), 1.27 (t, *J* = 7.1 Hz, 3H), 1.08 (t, *J* = 7.5 Hz, 3H); **^{13}C NMR** (101 MHz, $CDCl_3$) δ 201.1, 166.9, 89.7, 82.5, 61.4, 49.3, 42.3, 14.1, 14.1, 13.3, 12.3; **HRMS** (ESI, negative ion mode) *m/z*: $[M - H]^-$ calculated for $C_{11}H_{15}O_3$: 195.1021; Found: 195.1028.

tert-Butyl 3-oxonon-6-ynoate (**11c**). Synthesized from *tert*-butyl acetoacetate according to the procedure for **11a**; purification with 5% ethyl acetate in hexanes, yellow oil, (795.0 mg, 35% based on 10 mmol of *tert*-butyl acetoacetate). **^{1}H NMR** (400 MHz, $CDCl_3$) δ 3.35 (s, 2H), 2.71 (t, *J* = 7.3 Hz, 2H), 2.41 (tt, *J* = 7.3, 2.4 Hz, 2H), 2.11 (qt, *J* = 7.5, 2.4 Hz, 2H), 1.45 (s, 9H), 1.07 (t, *J* = 7.5 Hz, 3H); **^{13}C NMR** (101 MHz, $CDCl_3$) δ 201.6, 166.2, 82.4, 82.0, 50.6, 42.3, 27.9, 14.1, 13.2, 12.3; **HRMS** (ESI, negative ion mode) *m/z*: $[M - H]^-$ calculated for $C_{13}H_{19}O_3$: 223.1334; Found: 223.1334.

Dimethyl (2-oxooct-5-yn-1-yl)phosphonate (**11d**). Synthesized from dimethyl (2-oxopropyl)phosphonate according to the procedure for **11a**; purification with 60% ethyl acetate in hexanes, orange oil, (566.0 mg, 48% based on 5 mmol of dimethyl (2-

oxopropyl)phosphonate). **^{1}H NMR** (400 MHz, $CDCl_3$) δ 3.74 (d, $^3J_{H\text{-}P}$ = 11.2 Hz, 6H), 3.07 (d, $^2J_{H\text{-}P}$ = 22.7 Hz, 2H), 2.77 (t, J = 7.2 Hz, 2H), 2.37 (tt, J = 7.2, 2.4 Hz, 2H), 2.08 (qt, J = 7.5, 2.4 Hz, 2H), 1.04 (t, J = 7.5 Hz, 3H); **^{13}C NMR** (101 MHz, $CDCl_3$) δ 200.2 (d, $^2J_{H\text{-}P}$ = 6.5 Hz), 82.4, 77.3, 53.0 (d, $^2J_{H\text{-}P}$ = 6.5 Hz), 43.4(d, $^3J_{H\text{-}P}$ = 1.4 Hz), 41.9 & 40.6 (d, J = 128.3 Hz), 14.1, 13.2, 12.3; **HRMS** (ESI, negative ion mode) *m/z*: $[M - H]^-$ calculated for $C_{10}H_{16}O_4P$: 231.0786; Found: 231.0788.

1-(Phenylsulfonyl)oct-5-yn-2-one (**11e**). Synthesized from phenylsulfonylacetone according to the procedure for **11a**; purification with 20% ethyl acetate in hexanes followed by 30% ethyl acetate in hexanes, beige solid, (1.09 g, 41% based on 10 mmol phenylsulfonylacetone). **Mp** 58.4–60.8 ˚C, **^{1}H NMR** (400 MHz, $CDCl_3$) δ 7.87 (d, J = 7.7 Hz, 2H), 7.67 (t, J = 7.7 Hz, 1H), 7.56 (t, J = 7.7 Hz, 2H), 4.17 (s, 2H), 2.90 (t, J = 7.1 Hz, 2H), 2.39 (tt, J = 7.1, 2.4 Hz, 2H), 2.10 (qt, J = 7.5, 2.4 Hz, 2H), 1.07 (t, J = 7.5 Hz, 3H); **^{13}C NMR** (101 MHz, $CDCl_3$) δ 196.6, 138.5, 134.3, 129.3, 128.3, 82.8, 76.9, 66.9, 43.6, 14.1, 13.2, 12.3; **HRMS** (ESI, negative ion mode) *m/z*: $[M - H]^-$ calculated for $C_{14}H_{15}O_3S$: 263.0742; Found: 264.0744.

IV. C/H Correlation spectrum for 9

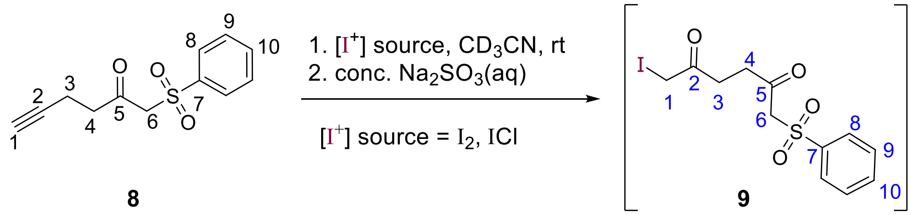

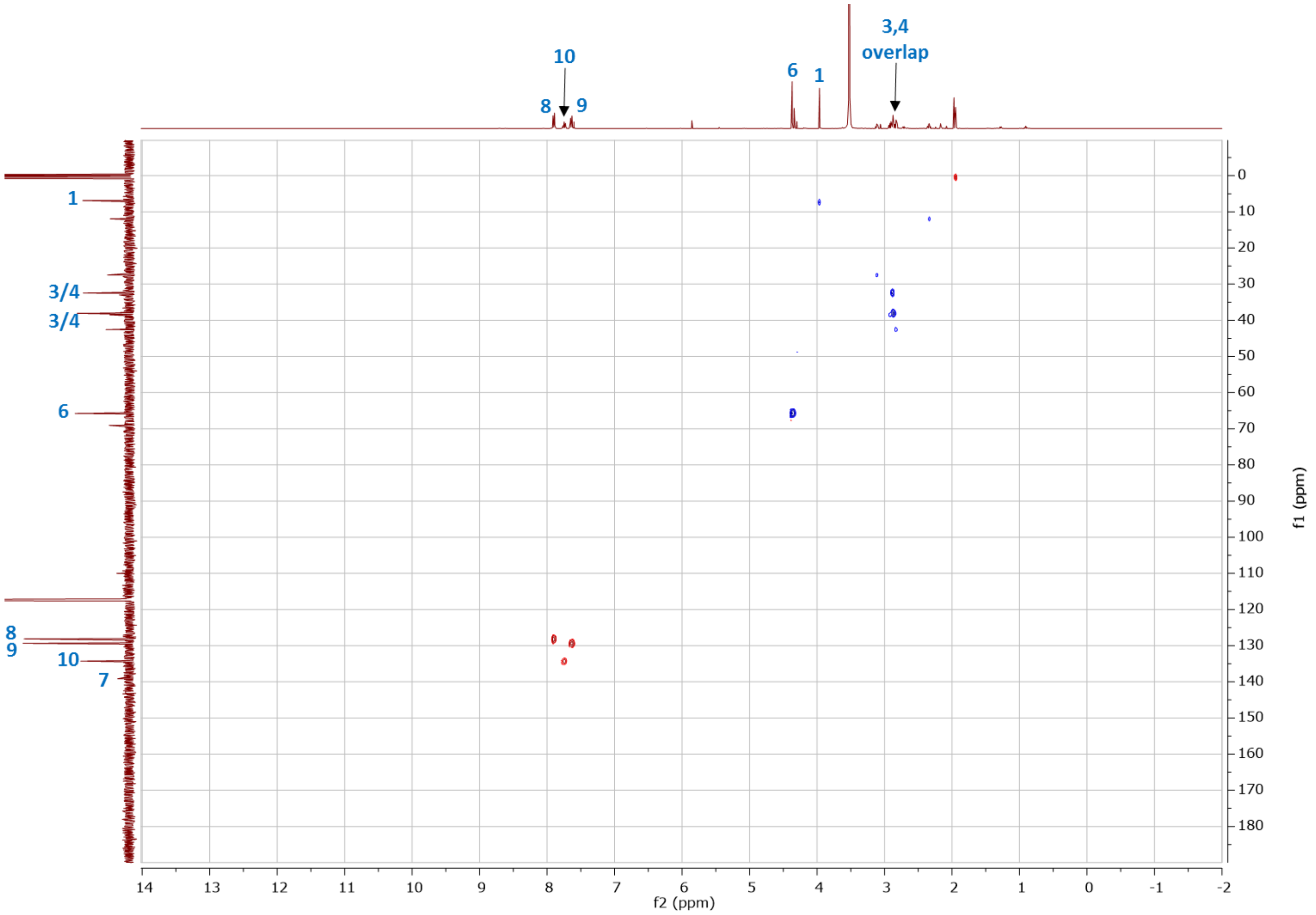

Figure S1: HSQC C/H Correlation spectra for α-iodo intermediate **9** in CD_3CN.

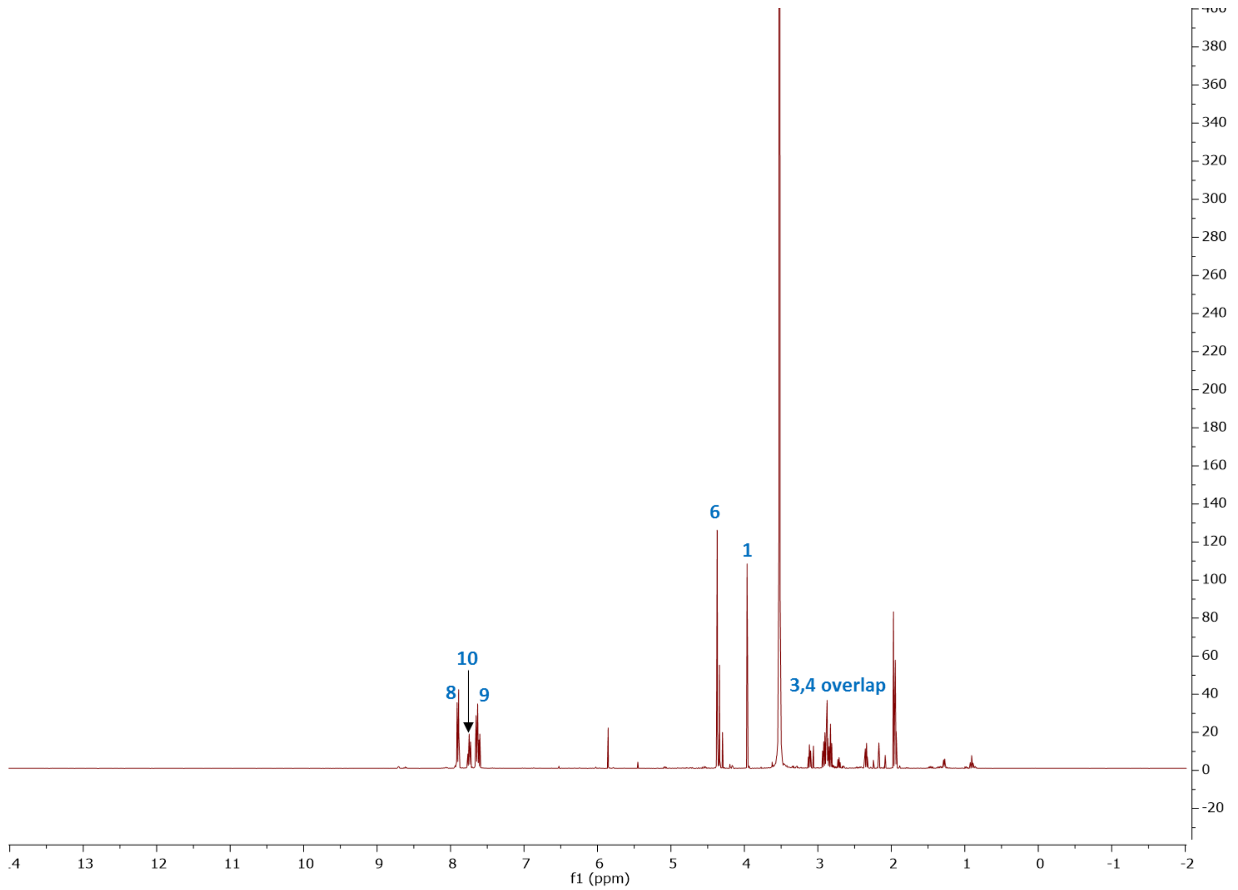

Figure S2: ^{1}H NMR Spectrum for for α-iodo intermediate **9** in CD_3CN

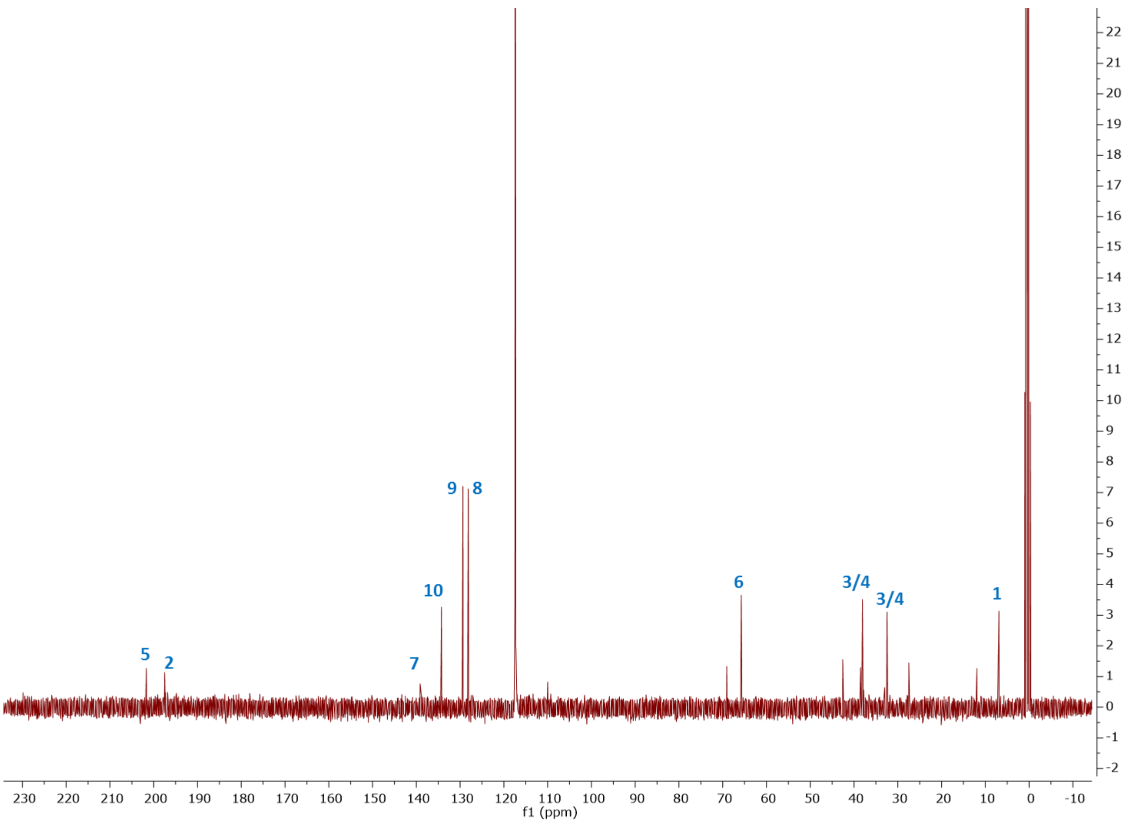

Figure S3: ^{13}C NMR for α-iodo intermediate **9** in CD_3CN

V. ^{1}H- and ^{13}C NMR spectra for substrates and hydration products

^{1}H NMR

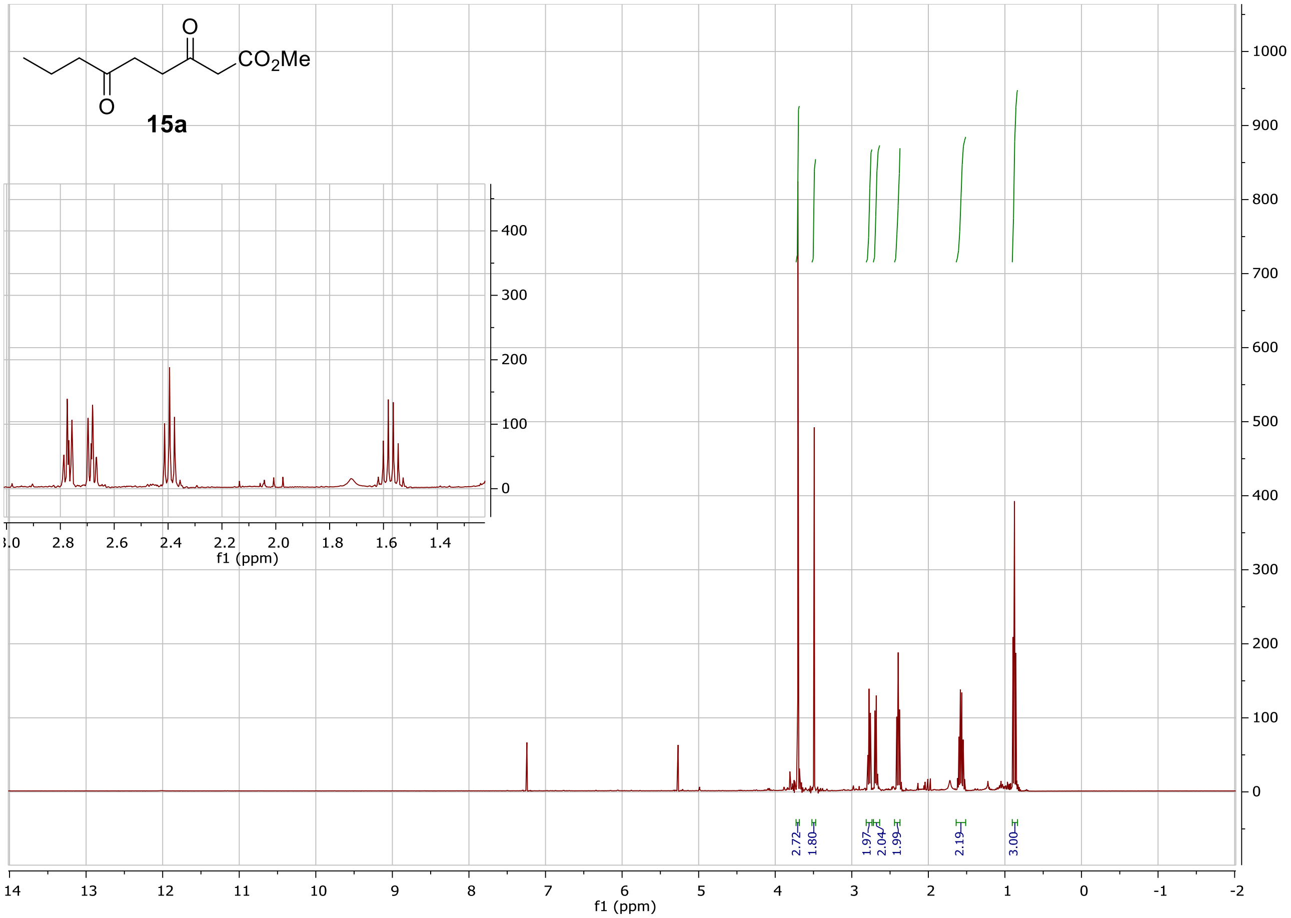

^{13}C NMR

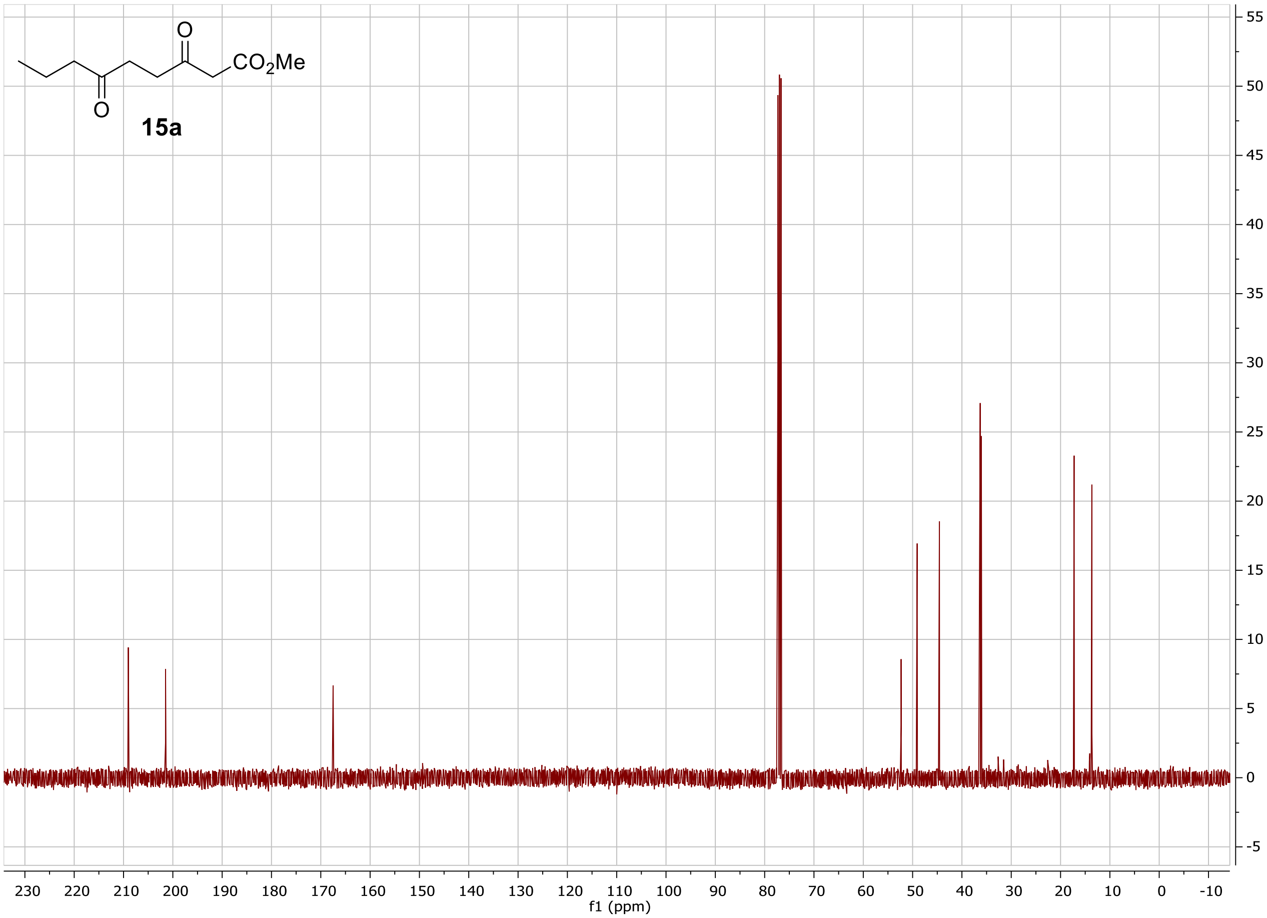

^{1}H NMR

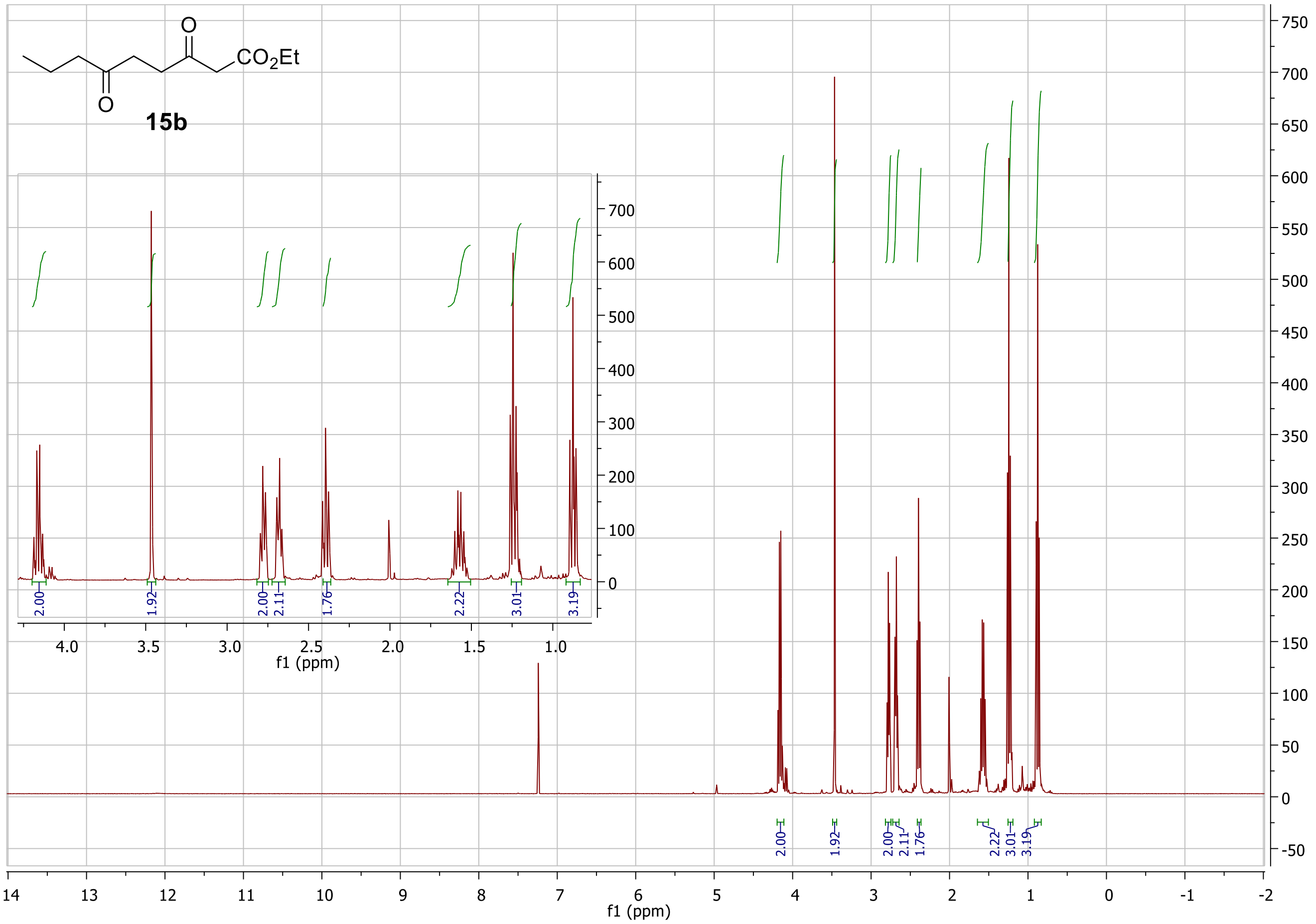

^{13}C NMR

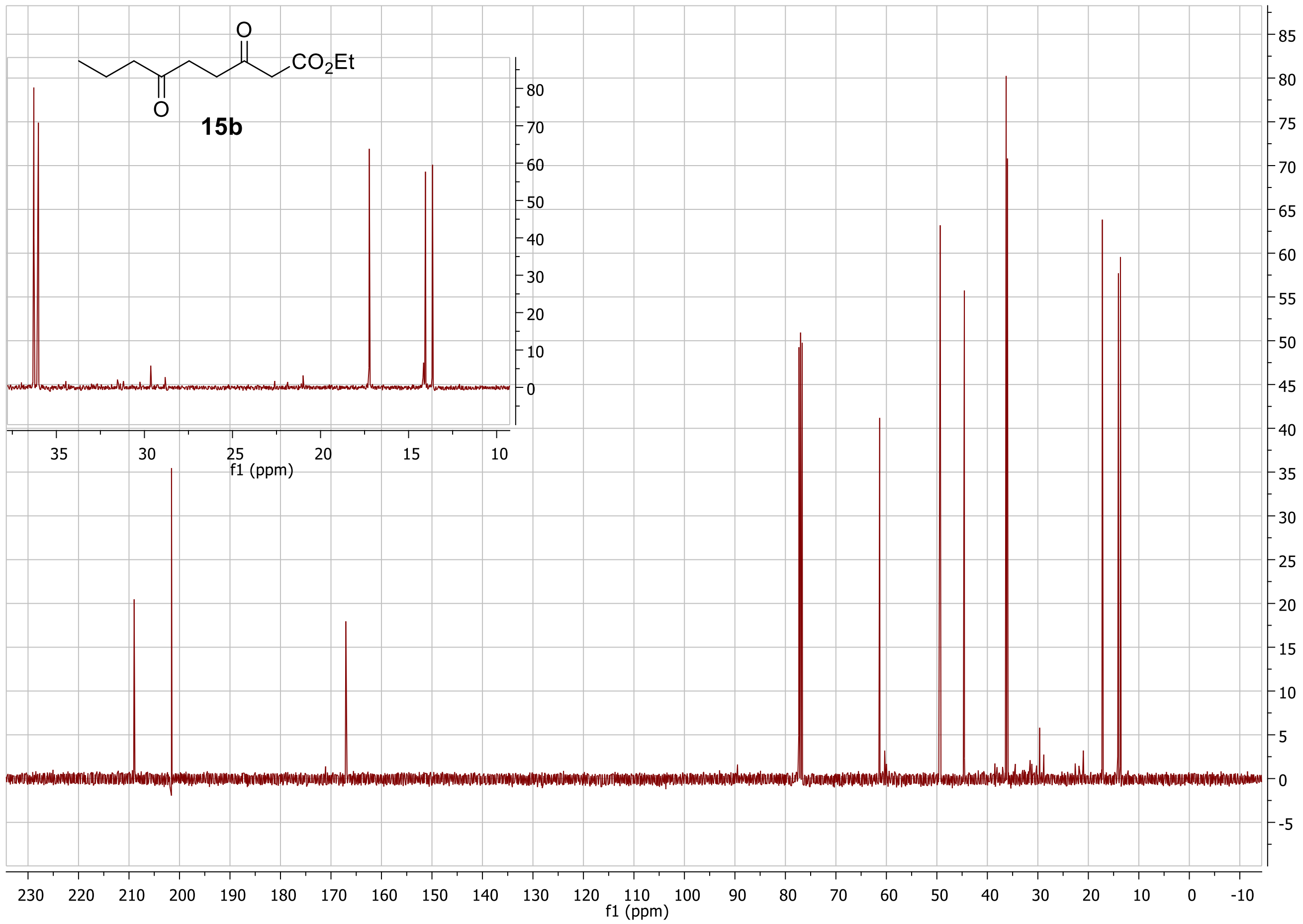

^{1}H NMR

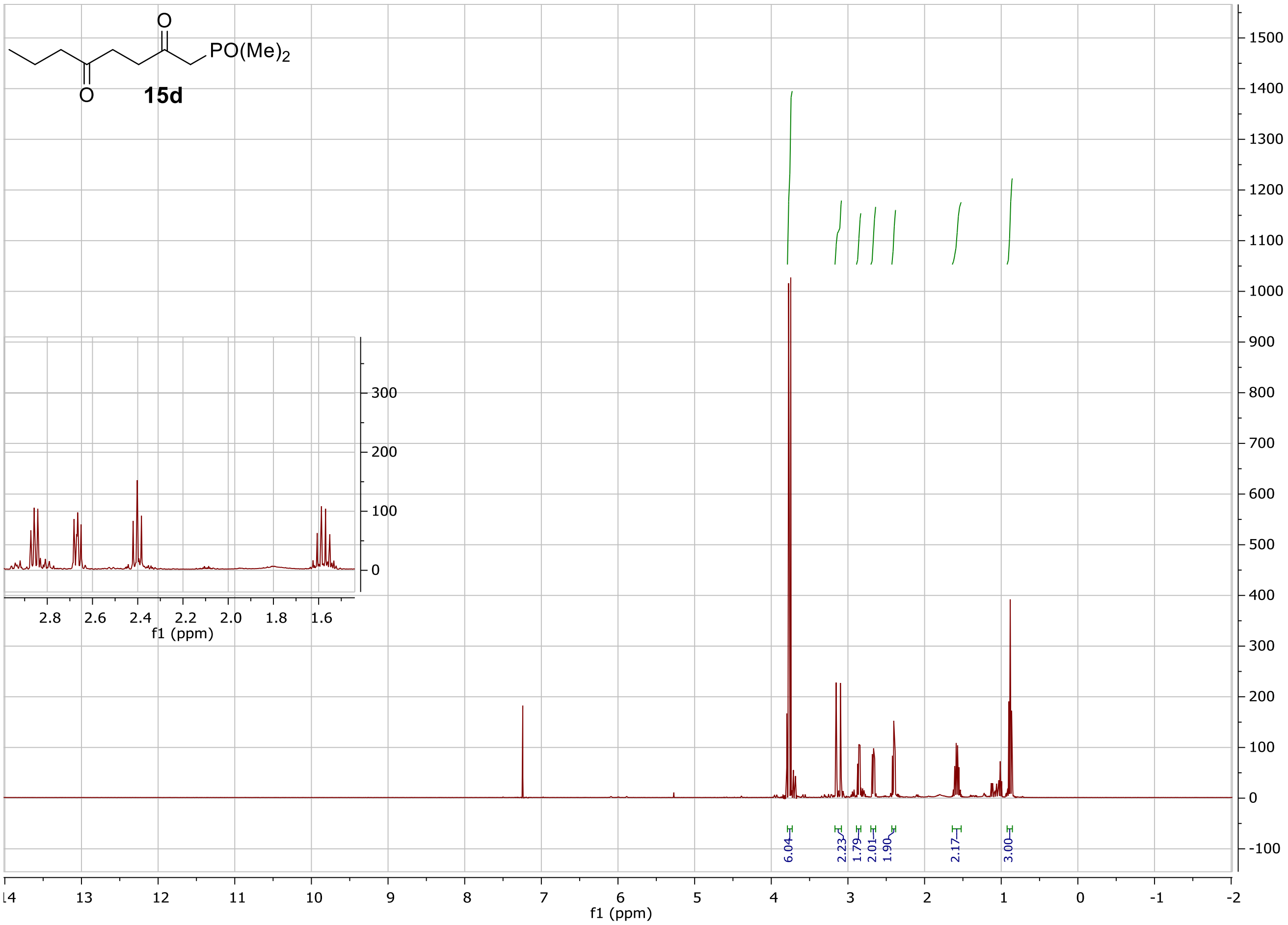

^{13}C NMR

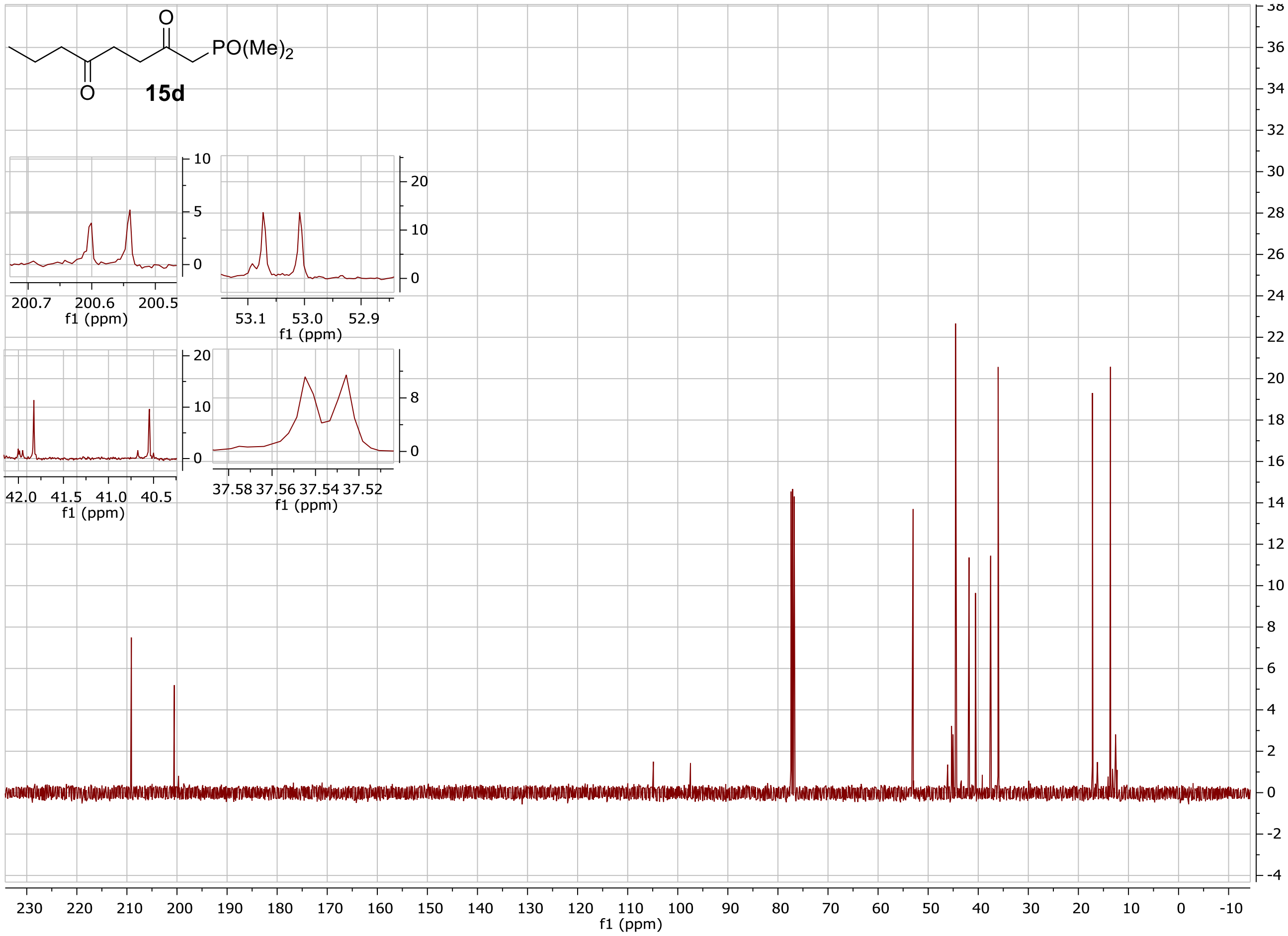

^{1}H NMR

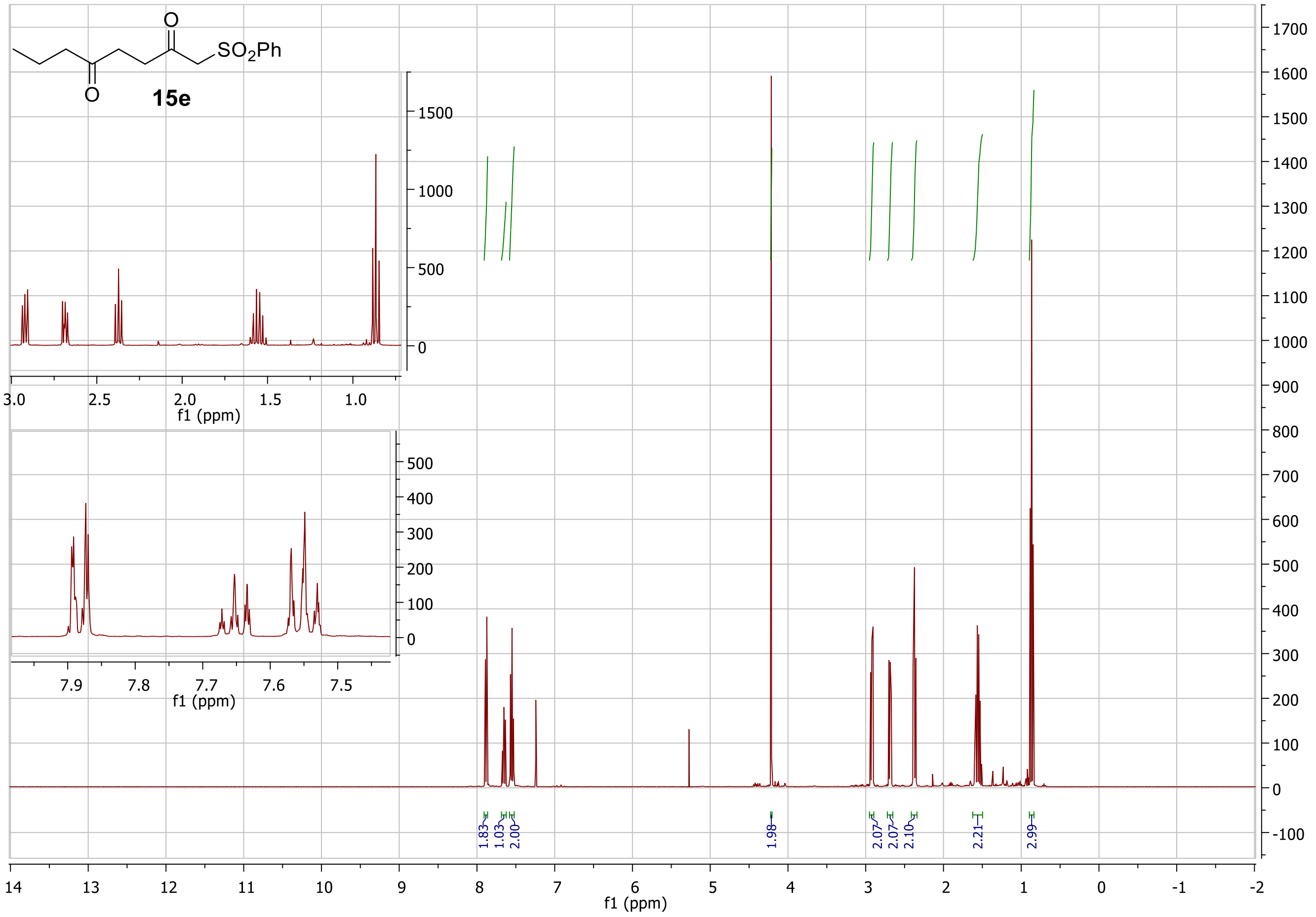

^{13}C NMR

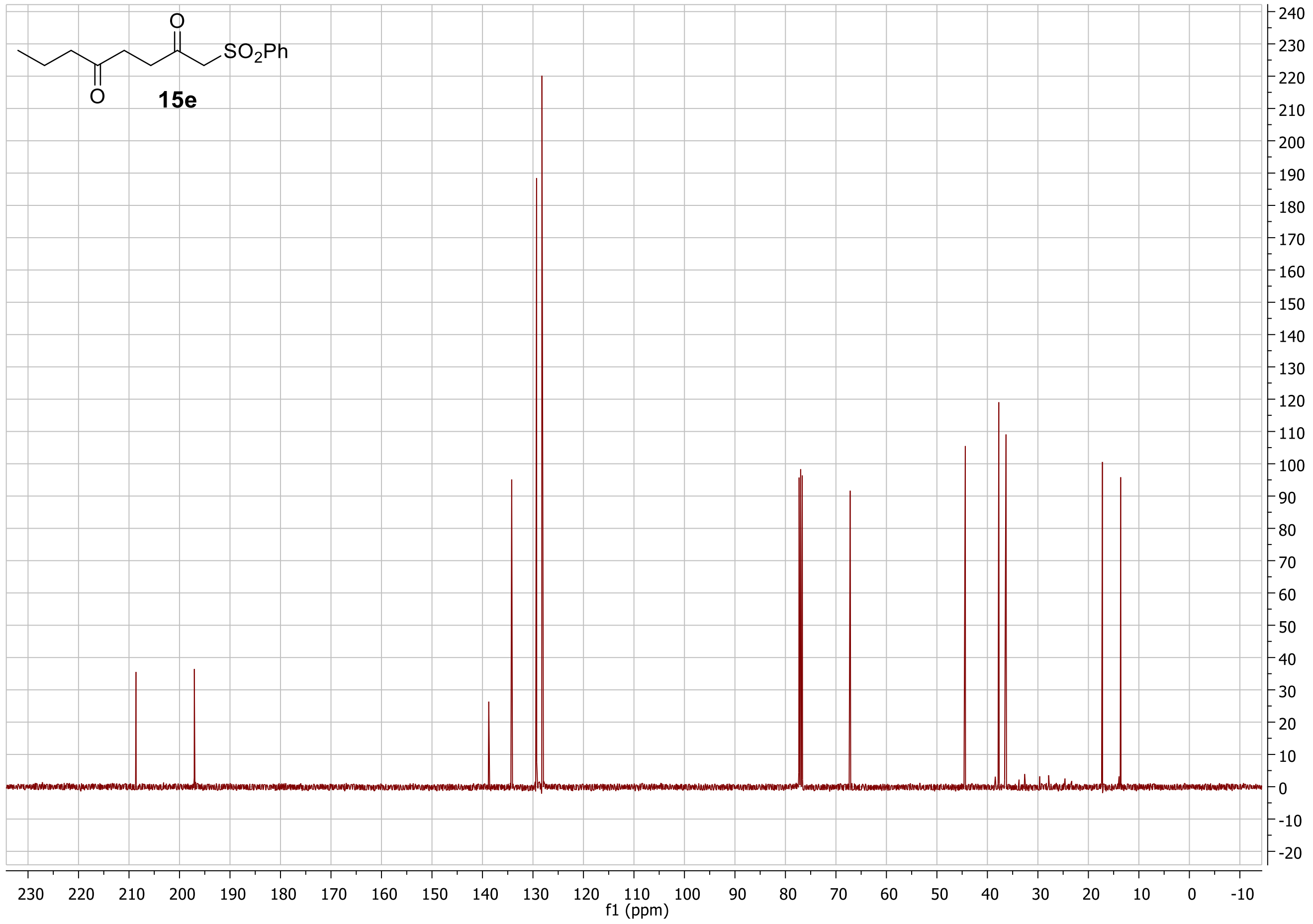

O
SO2Ph
O
15e
240
230
220
210
200
190
180
170
160
150
140
130
120
110
100
90
80
70
60
50
40
30
20
10
0
-10
-20
230 220 210 200 190 180 170 160 150 140 130 120 110 100 90 80 70 60 50 40 30 20 10 0 -10
f1 (ppm)

^{1}H NMR

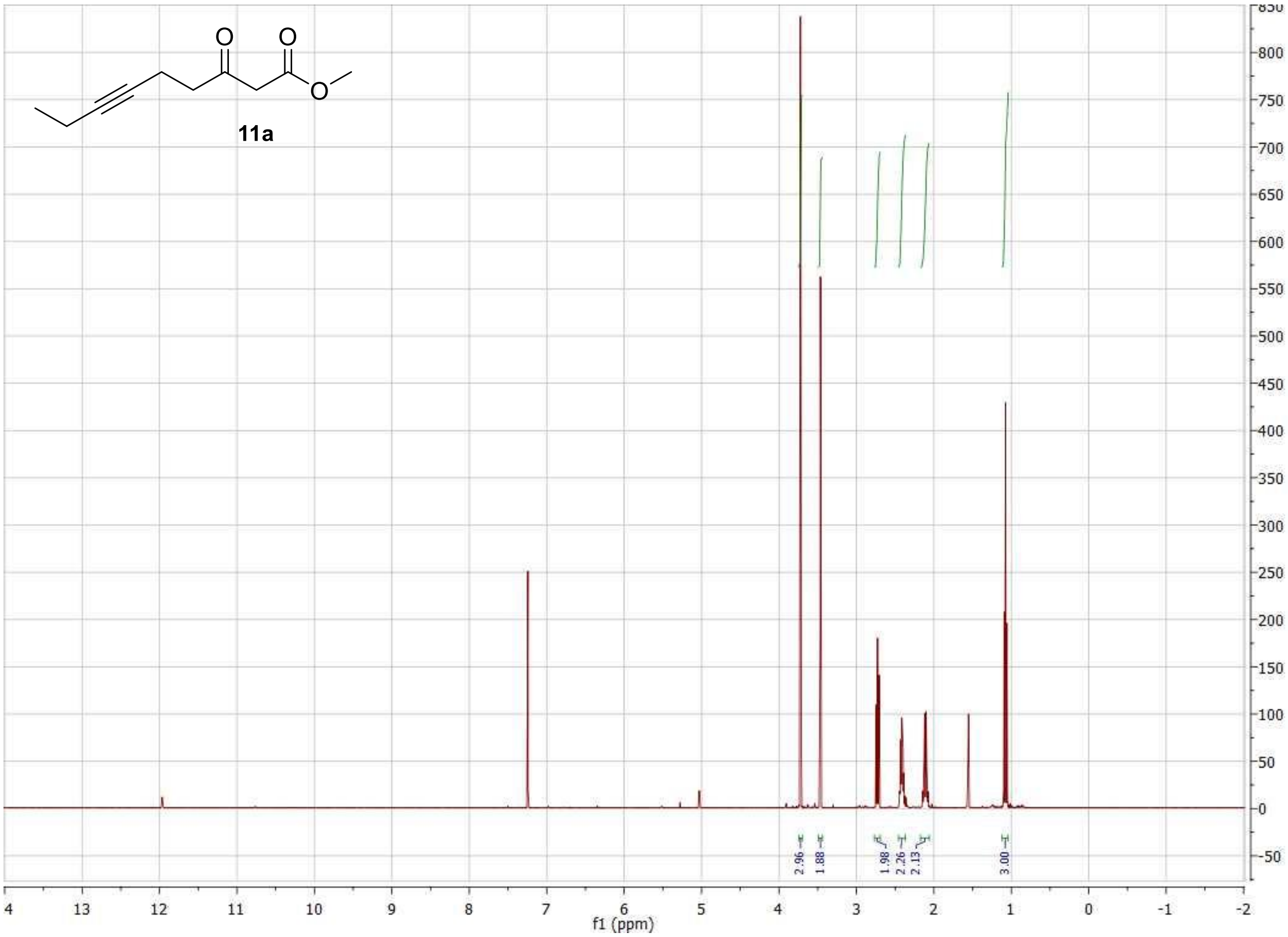

^{13}C NMR

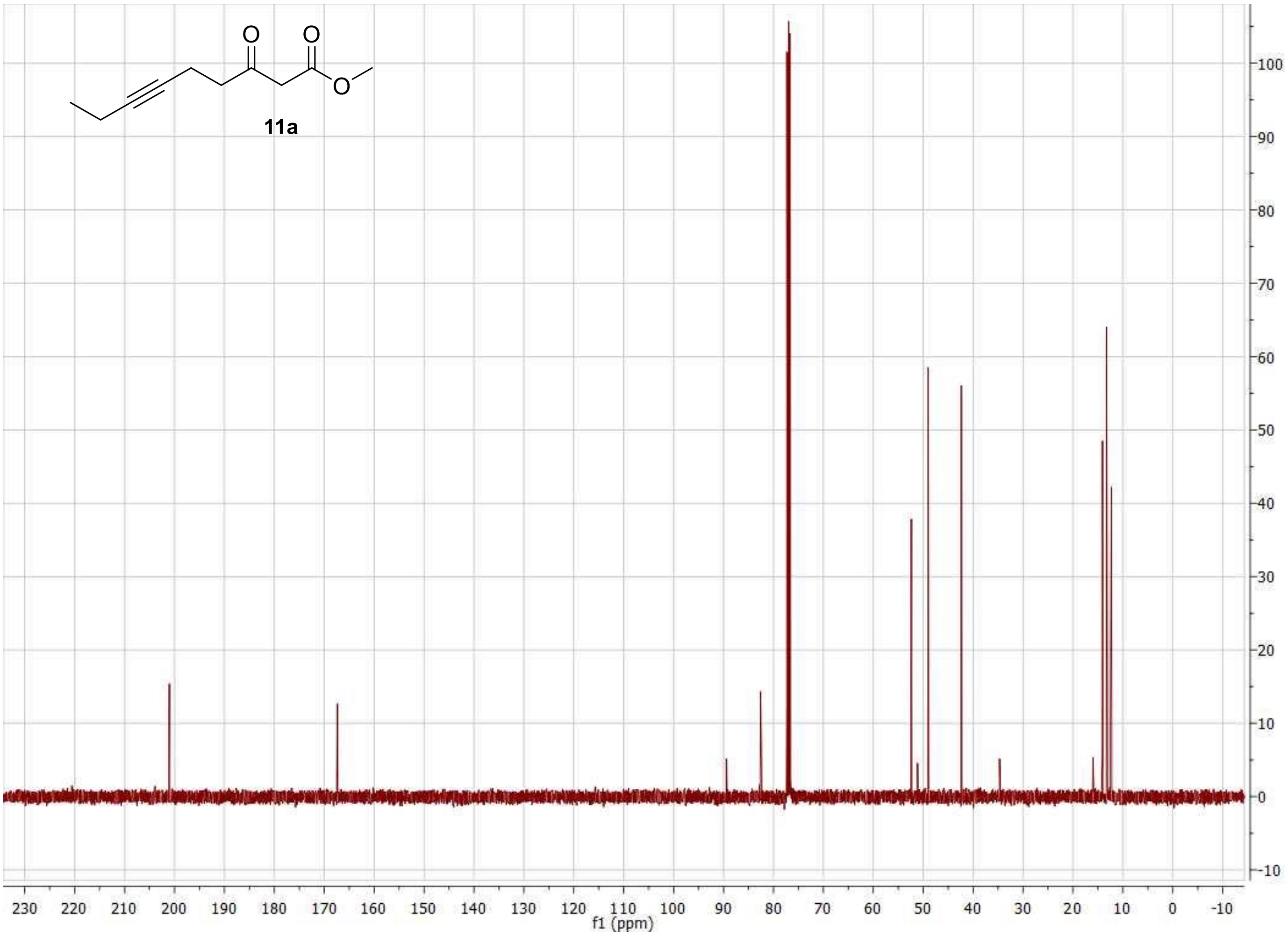

^{1}H NMR

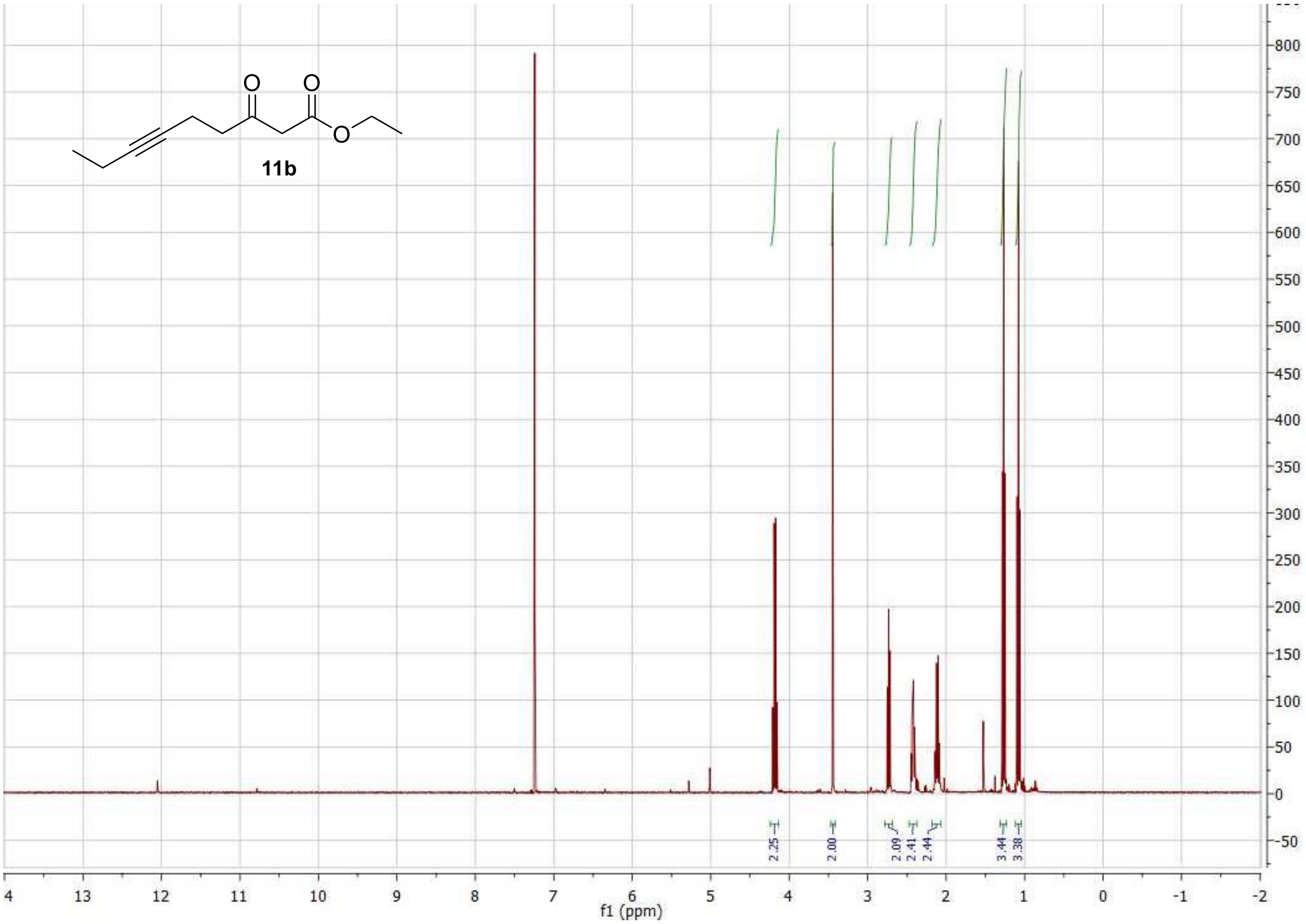

^{13}C NMR

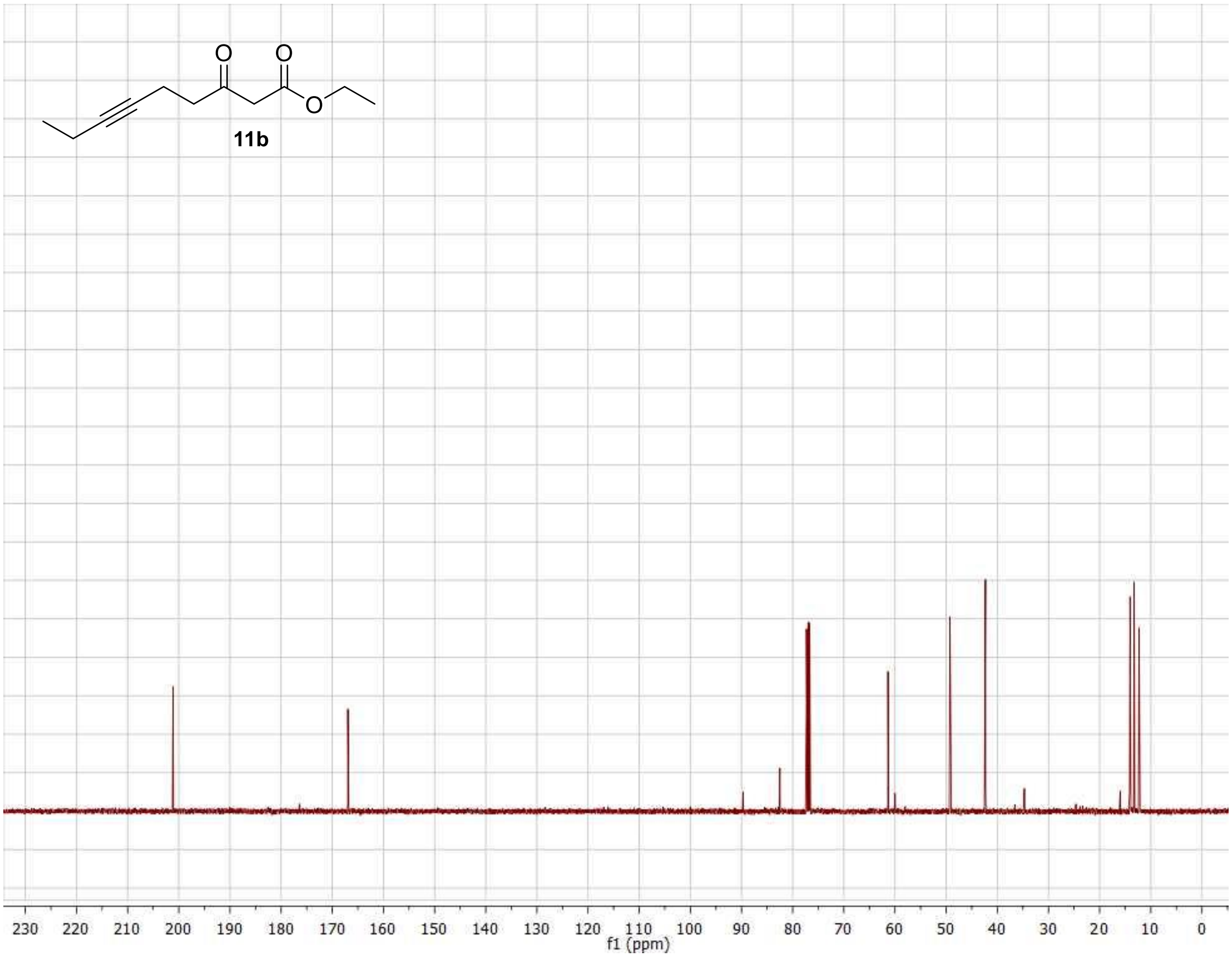

11b
230
220
210
200
190
180
170
160
150
140
130
120
110
100
90
80
70
60
50
40
30
20
10
0
f1 (ppm)

^{1}H NMR

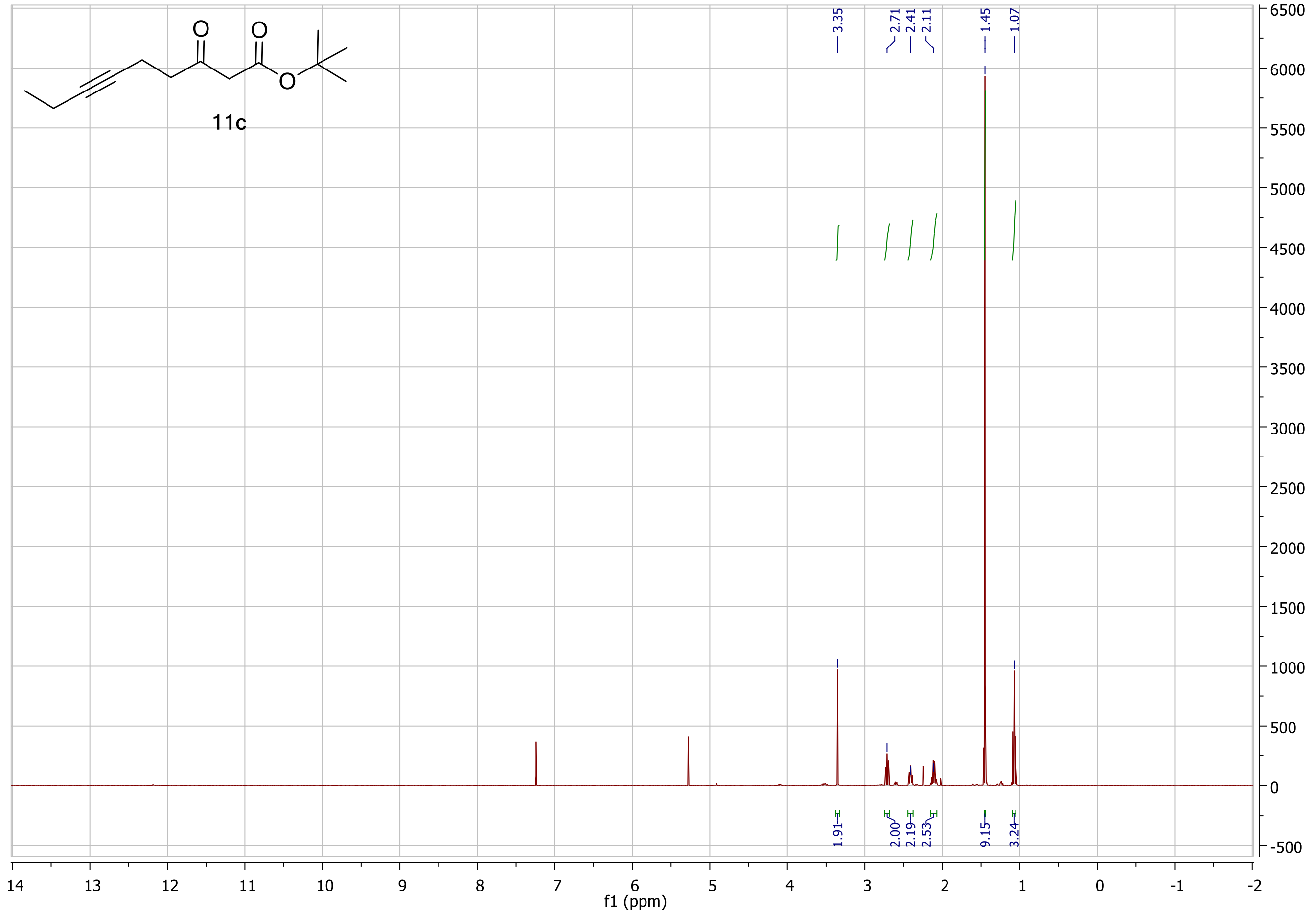

^{13}C NMR

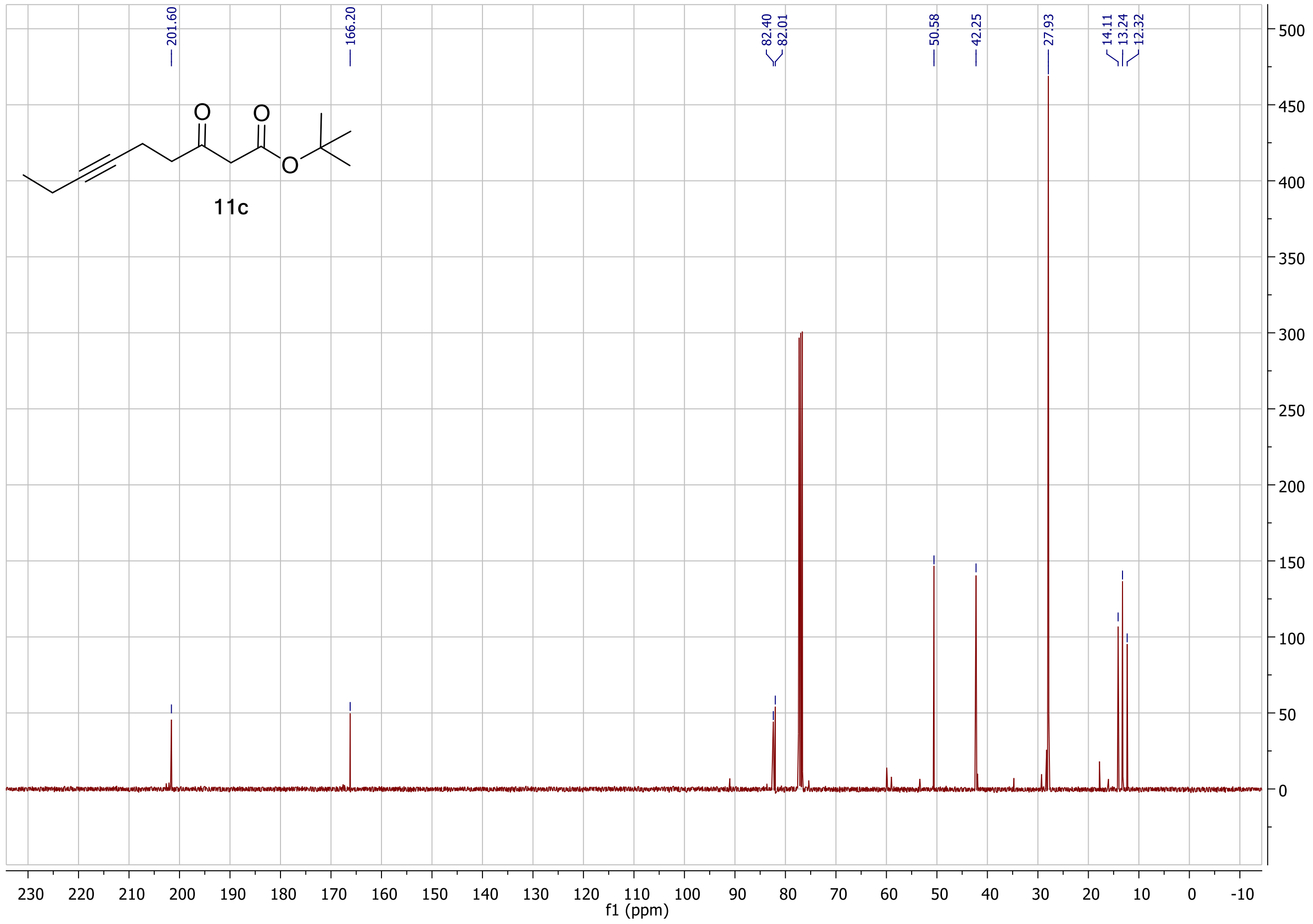

201.60
166.20
82.40
82.01
50.58
42.25
27.93
14.11
13.24
12.32
11c
f1 (ppm)

^{1}H NMR

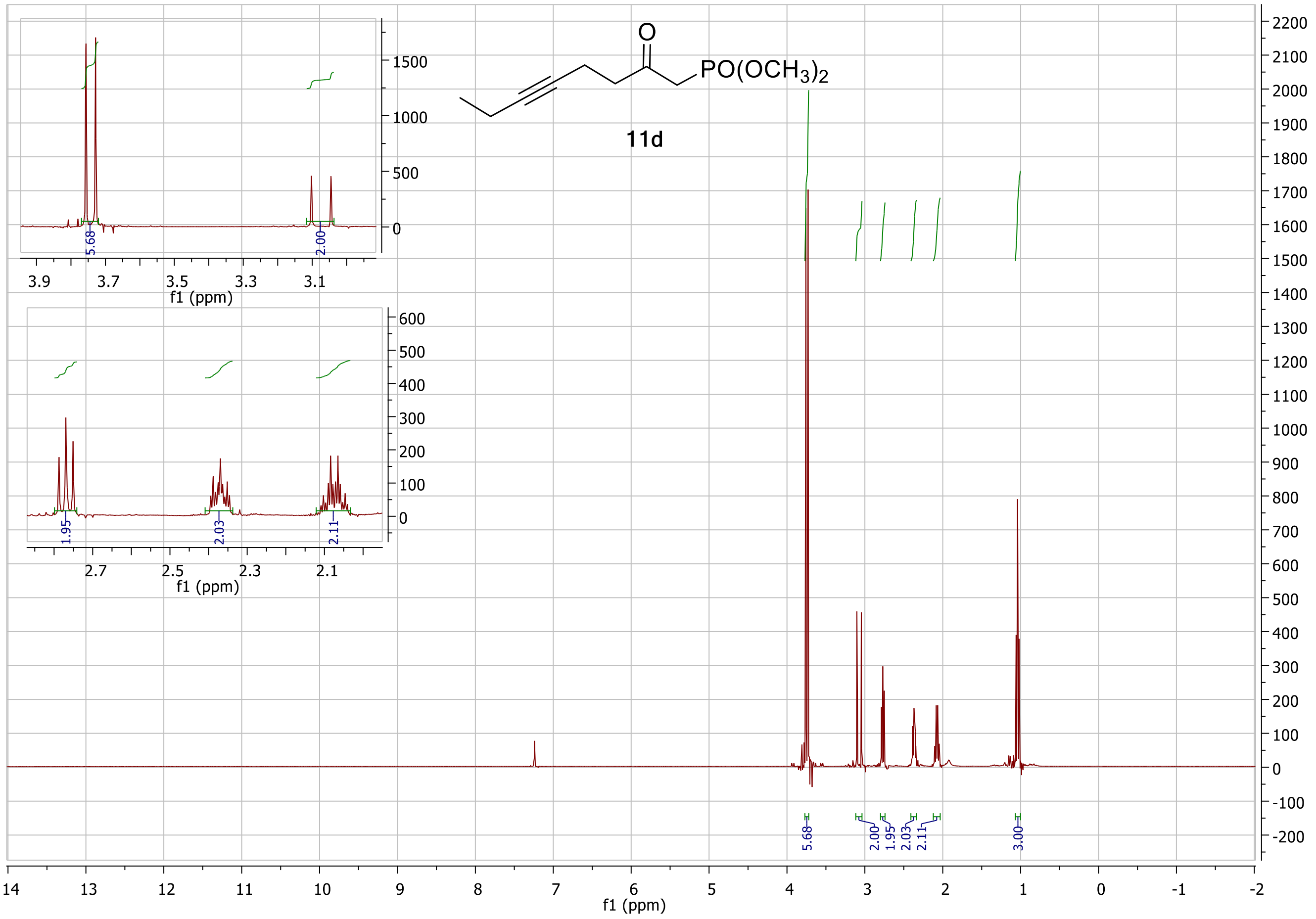

^{13}C NMR

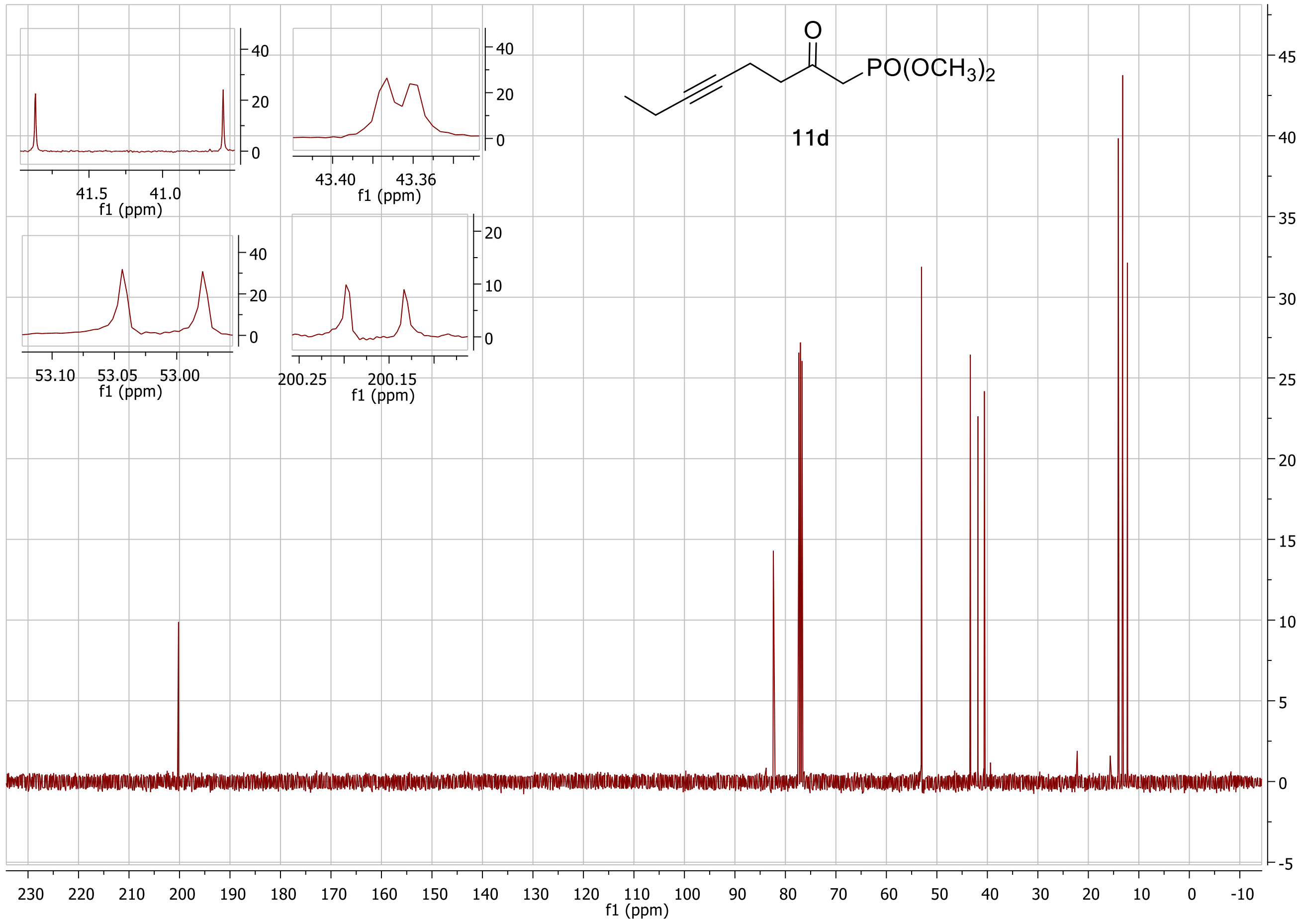

^{1}H NMR

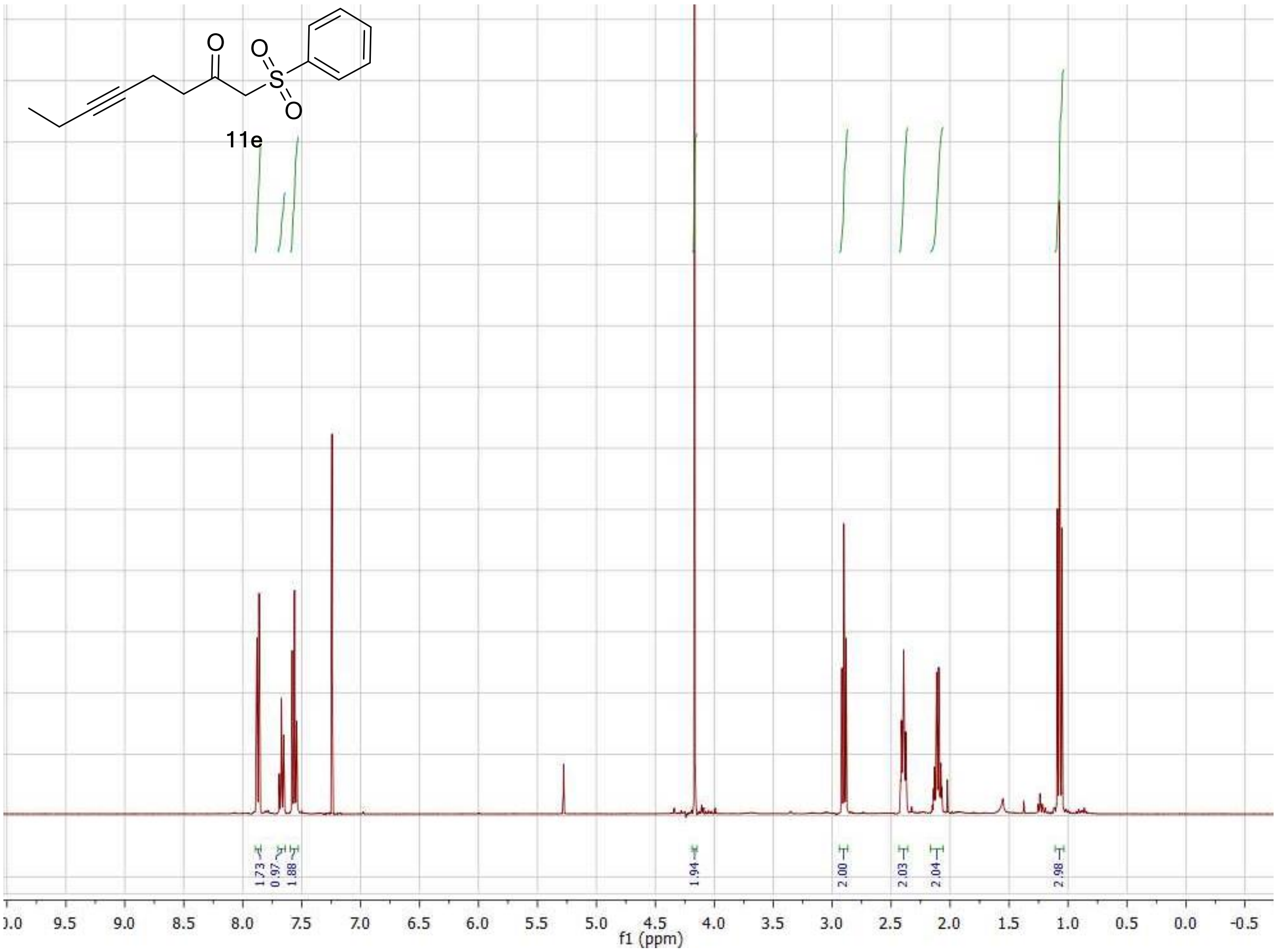

^{13}C NMR

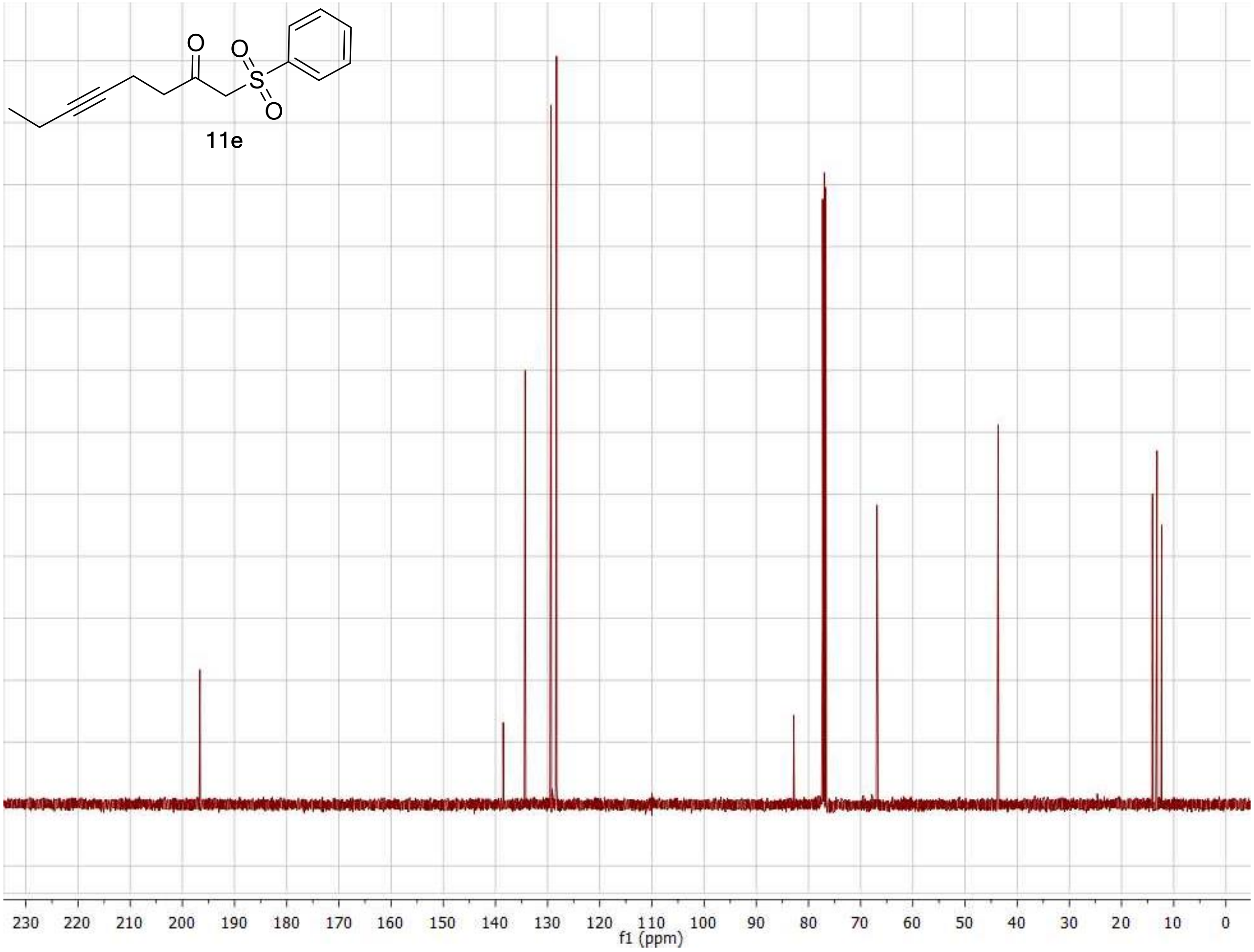

11e
230 220 210 200 190 180 170 160 150 140 130 120 110 100 90 80 70 60 50 40 30 20 10 0
f1 (ppm)

VI. High resolution mass spectra (negative ion mode)

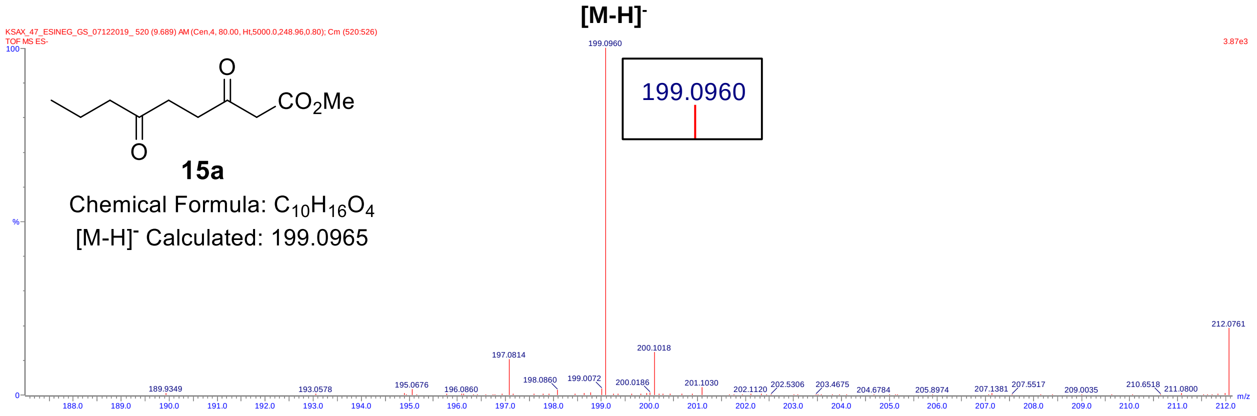

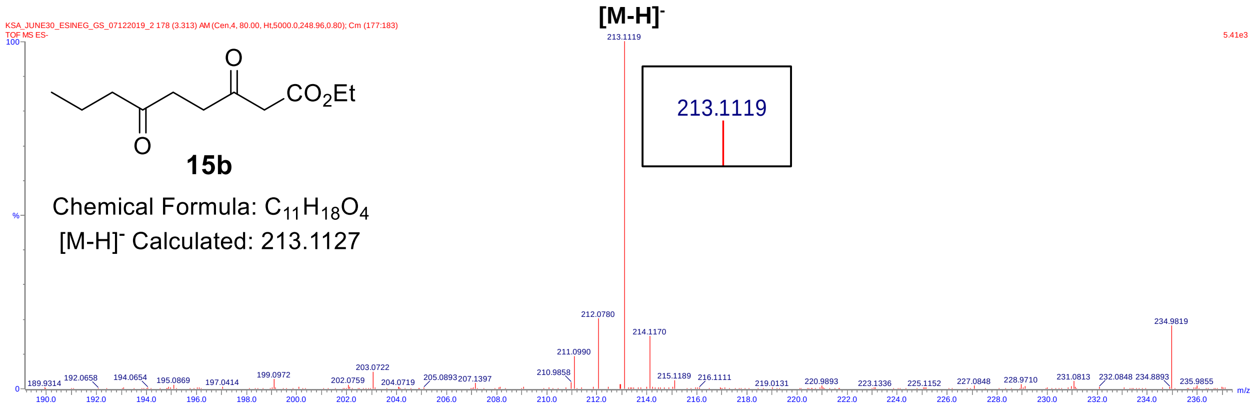

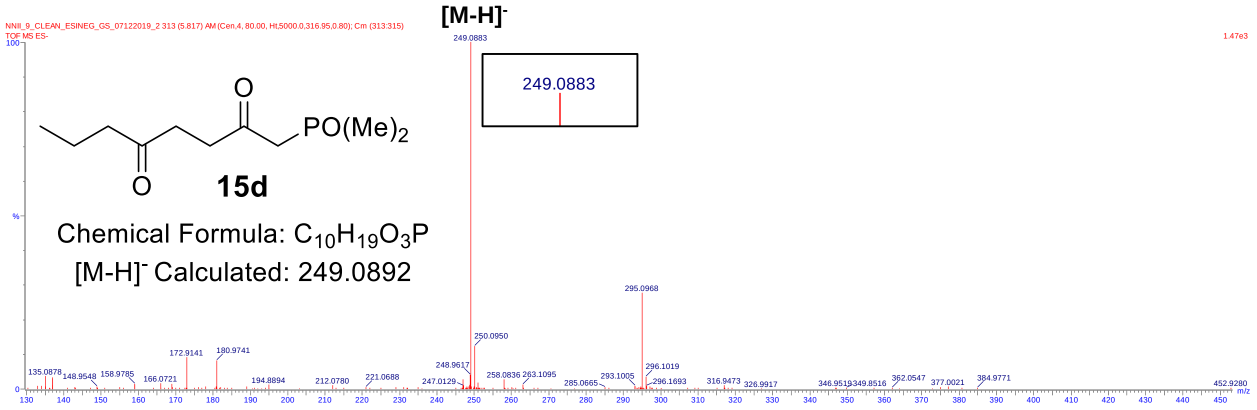

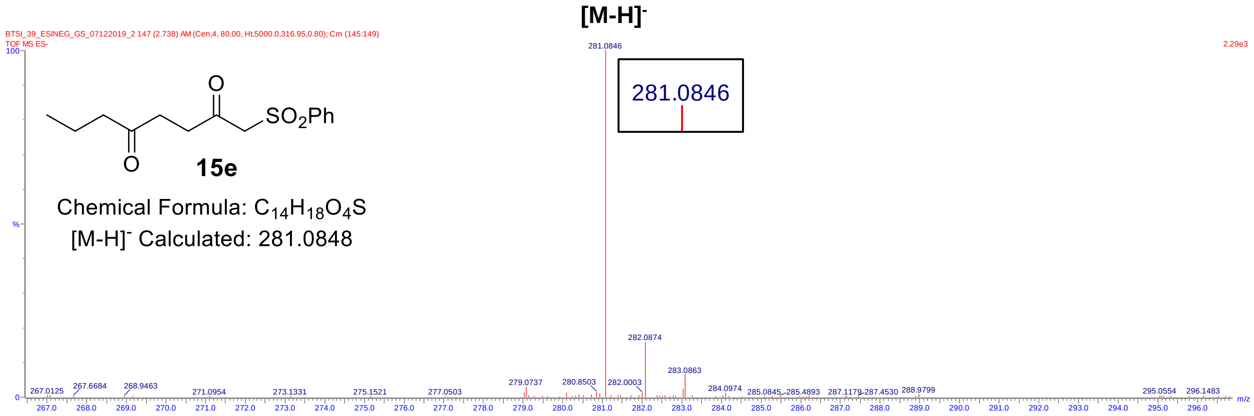
[M-H]-
BTSI_39_ESINEG_GS_07122019_2 147 (2.738) AM (Cen,4, 80.00, Ht,5000.0,316.95,0.80); Cm (145:149)
TOF MS ES-
281.0846
SO2Ph
15e
Chemical Formula: C14H18O4S
[M-H]- Calculated: 281.0848
282.0874
283.0863
2.29e3
m/z

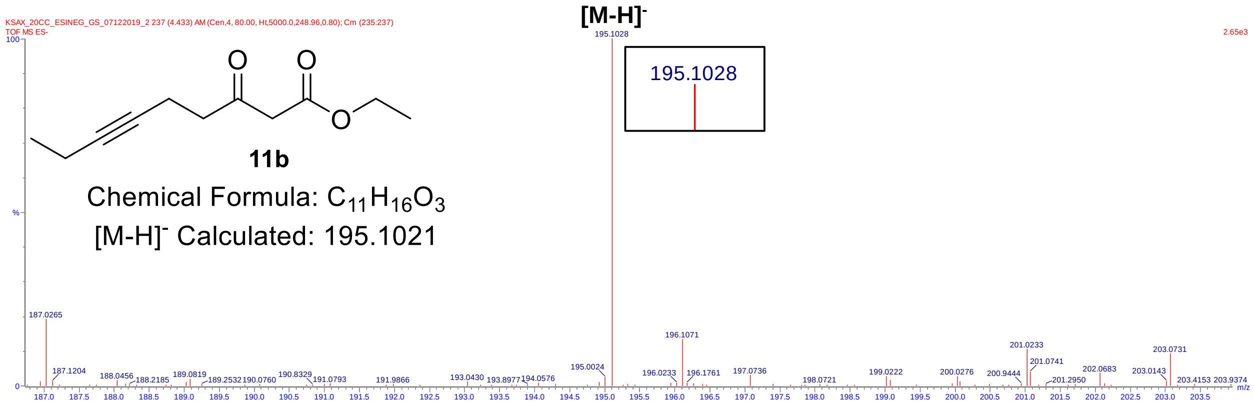
[M-H]-
KSAX_20CC_ESINEG_GS_07122019_2 237 (4.433) AM (Cen,4, 80.00, Ht,5000.0,248.96,0.80); Cm (235:237)
TOF MS ES-
195.1028
11b
Chemical Formula: C11H16O3
[M-H]- Calculated: 195.1021
187.0265
196.1071
201.0233
203.0731
2.65e3
m/z

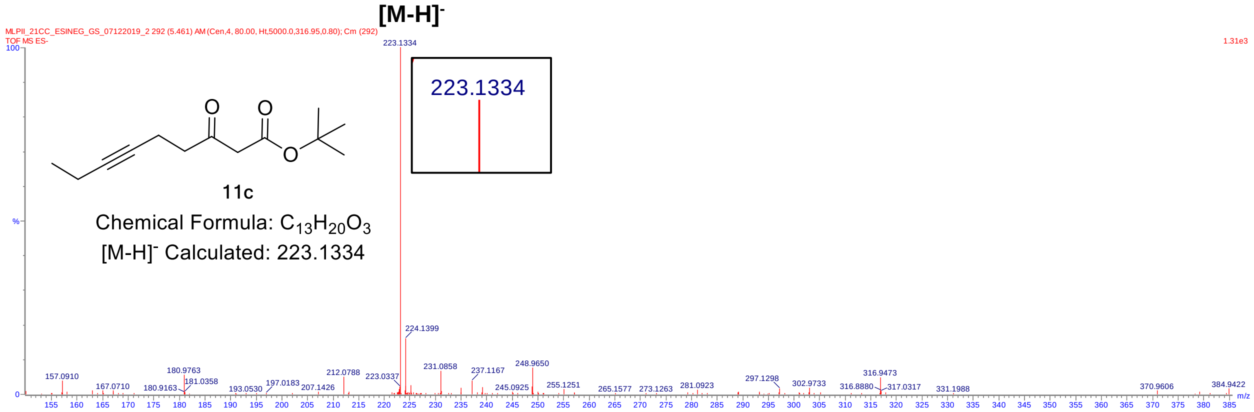
[M-H]-
MLPII_21CC_ESINEG_GS_07122019_2 292 (5.461) AM (Cen,4, 80.00, Ht,5000.0,316.95,0.80); Cm (292)
TOF MS ES-
223.1334
11c
Chemical Formula: C13H20O3
[M-H]- Calculated: 223.1334
224.1399
1.31e3
m/z

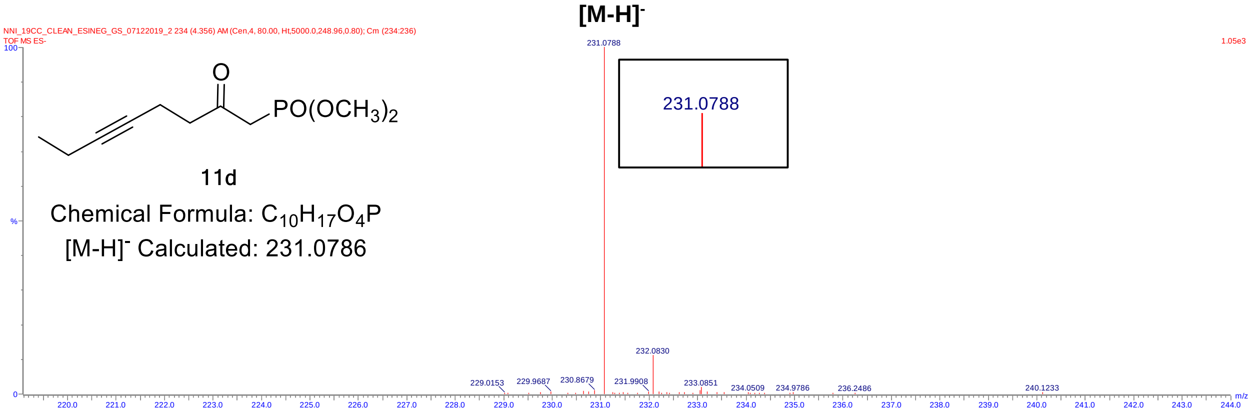

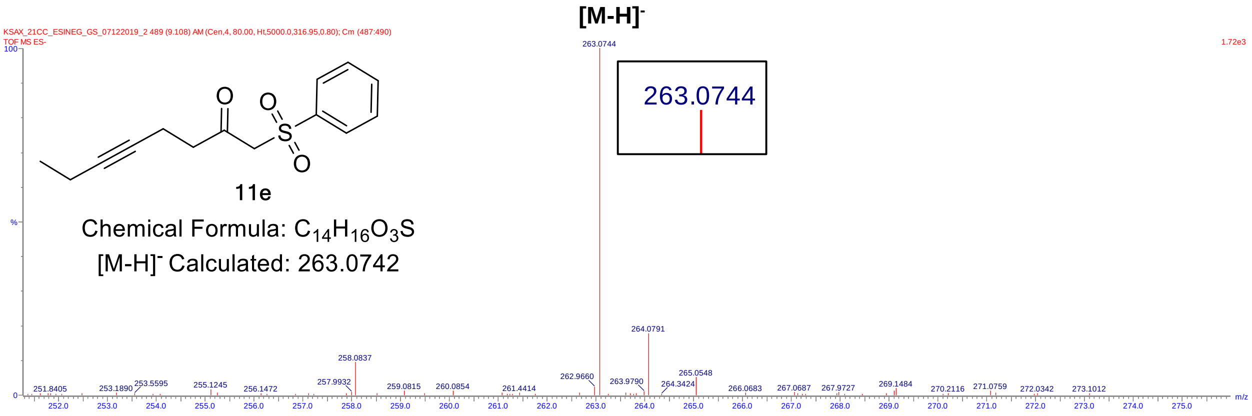

References

1. Achmatowicz, O., Jr.; Cynkowski, T.; Bukowski, P. *Polish Journal of Chemistry* **1983**, *57* (7-8-9), 1047-50.
